# Metabolomics approach reveals high energy diet improves the quality and enhances the flavor of black Tibetan sheep meat by altering the composition of rumen microbiota

**DOI:** 10.3389/fnut.2022.915558

**Published:** 2022-08-10

**Authors:** Xue Zhang, Lijuan Han, Shengzhen Hou, Sayed Haidar Abbas Raza, Linsheng Gui, Shengnan Sun, Zhiyou Wang, Baochun Yang, Zhenzhen Yuan, Jesus Simal-Gandara, Ahmed M. El-Shehawi, Amal Alswat, Muneefah A. Alenezi, Mustafa Shukry, Samy M. Sayed, Bandar Hamad Aloufi

**Affiliations:** ^1^College of Agriculture and Animal Husbandry, Qinghai University Xining, Xining, China; ^2^College of Animal Science and Technology, Northwest A&F University, Yangling, Shaanxi, China; ^3^Nutrition and Bromatology Group, Department of Analytical Chemistry and Food Science, Faculty of Food Science and Technology, University of Vigo—Ourense Campus, Ourense, Spain; ^4^Department of Biotechnology, College of Science, Taif University, Taif, Saudi Arabia; ^5^Department of Biology, Faculty of Science, University of Tabuk, Tabuk, Saudi Arabia; ^6^Department of Physiology, Faculty of Veterinary Medicine, Kafrelsheikh University, Kafr El Sheikh, Egypt; ^7^Department of Science and Technology, University College-Ranyah, Taif University, Taif, Saudi Arabia; ^8^Department of Biology, College of Science, University of Hail, Ha'il, Saudi Arabia

**Keywords:** black Tibetan sheep, dietary energy levels, meat quality, metabolomics, rumen microbiota

## Abstract

This study aims to determine the impact of dietary energy levels on rumen microbial composition and its relationship to the quality of Black Tibetan sheep meat by applying metabolomics and Pearson's correlation analyses. For this purpose, UHPLC-QTOF-MS was used to identify the metabolome, whereas 16S rDNA sequencing was used to detect the rumen microbiota. Eventually, we observed that the high energy diet group (HS) improved the carcass quality of Black Tibetan sheep and fat deposition in the *longissimus lumborum* (LL) compared to the medium energy diet group (MS). However, HS considerably increased the texture, water holding capacity (WHC), and volatile flavor of the LL when compared to that of MS and the low energy diet group (LS). Metabolomics and correlation analyses revealed that dietary energy levels mainly affected the metabolism of carbohydrates and lipids of the LL, which consequently influenced the content of volatile flavor compounds (VOCs) and fats. Furthermore, HS increased the abundance of *Quinella, Ruminococcus 2, (Eubacterium) coprostanoligenes*, and *Succinivibrionaceae UCG-001*, all of which participate in the carbohydrate metabolism in rumen and thus influence the metabolite levels (stachyose, isomaltose, etc.) in the LL. Overall, a high-energy diet is desirable for the production of Black Tibetan sheep mutton because it improves the mouthfeel and flavor of meat by altering the composition of rumen microbiota, which influences the metabolism in the LL.

## Introduction

Mutton from Black Tibetan sheep is a popular delicacy and a geographical indication product in China, mainly in Guinan County, Qinghai Province. Black Tibetan sheep mutton is known for its good palatability, pleasing aroma, and high nutrient value (1). However, the increase in population and consciousness of healthy living has increased the demand for more and better quality mutton. Furthermore, increased dietary energy increases sheep growth performance and carcass quality in intensive production (2). The dietary energy level affects the physical and chemical characteristics of meat and, therefore, its quality (3). Jiao et al. found that high-energy diets improved the carcass parameters (dressing percentage and eye muscle area) and meat quality (protein deposition and fatty acid composition) (1). Nevertheless, other studies suggest that the dietary energy level has no effect on the sensory attributes and physical or chemical properties of meat (4, 5). Meanwhile, the optimal dietary energy level for the best quality Black Tibetan sheep mutton is not known.

Muscle metabolites have been found to influence the physiological features and qualitative aspects of muscles as phenotypic components (6). Metabolomics investigations can reveal the whole spectra of compounds associated with distinct meat quality characteristics (7) because they directly and precisely represent the physiological condition of the organism (8). Diets affect the tenderness of Nellore cattle beef, which is related to the alteration of the levels of specific metabolites, including inosine monophosphate (IMP), carnosine, conjugated linoleic acid, and creatine in the longissimus thoracis (9). We found stall-feeding affected the protein and fat content in mutton by upregulating protein digestion and absorption pathway and downregulating lipolysis in adipocytes pathway of muscles in the previous research (10). Besides, Huang et al. reported that the differential fatty acids (FAs), amino acids (AAs), and glycogen metabolites are the key factors affecting the yak meat flavor (11). However, no research has been carried out for the analysis of varied dietary energy levels' effect on the metabolism of Black Tibetan sheep's LL.

The interaction between the host and rumen microbiota affects the metabolite deposition in muscles (12). Also, diet is a critical factor influencing the composition and function of rumen microbiota in ruminants (13). The changes in the rumen environment caused by different diets modify the morphology of rumen tissues, fermentation patterns, and microbial metabolism, which in turn affects the deposition of the compounds in muscles. For instance, feeding sheep with cactus as roughage significantly increases the abundance of *Butyrivibrio* and *Mogibacterium* in the animal's rumen (14). Abundant *Butyrivibrio* reduces the deposition of polyunsaturated fatty acids (PUFAs) in mutton (15). Meanwhile, a related study showed that the AA deposition in mutton was associated with the abundance of *Schwartzia* and *Moryella* in the rumen (12). In the present study, we hypothesized that altering the microbiota composition microbiota in Black Tibetan sheep rumen may have effects on muscle metabolism and meat quality.

The relationship between dietary energy levels and muscle metabolism along with its impact on the edible value, nutritional components, and volatile flavor of Black Tibetan sheep meat is largely unexplored yet. Moreover, changes in the abundance and diversity of rumen microbiota and its relationship with the quality of Black Tibetan sheep meat are not well understood. Thus, muscle metabolome and rumen microbiota composition were analyzed using UHPLC-QTOF-MS and Illumina-Miseq platform, respectively. The significant impact of dietary energy levels on rumen microbiota composition and its relationship with muscle metabolism and the subsequent effect on the quality and flavor of mutton based on constitutive metabolites were also evaluated. The findings of this study will reveal the optimal dietary energy level for producing the best quality Black Tibetan sheep mutton, thus promoting good development of the animal husbandry of Black Tibetan sheep.

## Materials and methods

The study was carried out at the Black Tibetan Sheep Breeding Center in Guinan County, Qinghai Province and was approved by the Animal Ethics Committee of Qinghai University (QUA-2020-0709).

### Animals, slaughter, and samples

Twenty-seven 120-day-old rams (Black Tibetan sheep) weighing 13.5 ± 0.78 kg were randomly assigned to three experimental groups with different energy level treatments, namely, high energy level (11.08 MJ/kg, HS, *n* = 9), medium energy level (10.12 MJ/kg, MS, *n* = 9), and low energy level (9.20 MJ/kg, LS, *n* = 9). Food was provided to each animal two times a day i.e., (08:00 and 18:00) within an individual unit (1.5 × 2.0 m^2^). After a 7-day adaption phase, the animals were subjected to a 120-day formal trial having *ad libitum* access to food and water. Table 1 shows the nutrients, components, and FA composition of three distinct diets given to animals. Furthermore, all animals were humanely slaughtered after solids fasting for 18 h and liquids fasting for 2 h at a local commercial slaughterhouse according to animal welfare procedures i.e., stun, exsanguination, skinning, evisceration, and removal of the head, feet, and tail. Both pre-slaughter weight (live body weight, LBW) and post-slaughter weight (hot carcass weight, HCW) of lambs were recorded. The LL from the left side of the carcasses between the 12th and 13th ribs were sampled after the evaluation of carcass quality and then, the color and initial pH value of each sample were determined within 45 min. Moreover, rumen fluid was collected in sterile containers after being filtered through four layers of cheesecloth. Professionals carried out the slaughter and sampling at the same time following standardized norms. All samples were frozen in liquid nitrogen before being stored at −80 °C in the laboratory for further analysis. For the assessment of meat quality and rumen fermentation characteristics, nine replicates of each group were employed, whereas muscle metabolomics and rumen microbiota studies were carried out on six replicates from each group, respectively.

**Table 1:** Dietary ingredients, nutrients, and fatty acid composition of three different energy level diets (dry matter basis).

**Items**	**HS group**	**MS group**	**LS group**
Dietary ingredients (% DM)			
Corn	42.28	30.18	16.22
Soybean meal	2.45	1.69	0.77
Rapeseed meal	12.96	7.15	3.92
Cottonseed meal	5.11	3.78	1.89
Oat silage	15.00	25.00	35.00
Oat hay	15.00	25.00	35.00
Mineral salt	0.80	0.80	0.80
Limestone	0.80	0.80	0.80
Baking soda	0.10	0.10	0.10
Dicalcium phosphate	0.50	0.50	0.50
Mineral/vitamin premix ^a^	5.00	5.00	5.00
Total	100.00	100.00	100.00
Nutritional levels (% DM)			
Digestive energy DE (MJ/kg)	11.08	10.12	9.20
Crude protein	13.25	12.83	12.47
Crude ash	4.53	5.22	6.06
Crude fat	5.13	4.32	3.55
Neutral detergent fiber NDF	26.38	36.47	46.54
Acid detergent fiber ADF	16.99	22.84	29.69
Calcium	0.87	0.83	0.79
Phosphorus	1.02	0.83	0.66
Fatty acid (mg/100 g DM)			
∑SFA	174.99	162.71	150.43
12:0	13.84	9.99	6.14
14:0	5.76	4.61	3.45
16:0	110.28	104.21	98.14
18:0	38.74	36.91	35.09
20:0	2.03	1.86	1.69
21:0	1.08	1.60	2.11
22:0	1.41	1.51	1.62
23:0	0.27	0.34	0.42
24:0	1.59	1.68	1.77
∑MUFA	134.06	118.46	102.87
c9-16:1	0.44	0.38	0.33
c9-18:1	102.94	91.38	79.82
c11-20:1	3.18	2.69	2.21
c13-22:1	26.48	23.00	19.51
c15-24:1	1.01	1.01	1.00
∑PUFA	191.39	184.78	178.17
18:2n-6	158.20	141.34	124.47
18:3n-6	0.11	0.18	0.26
18:3n-3	29.94	40.08	50.23
20:2n-6	0.58	0.47	0.37
20:3n-3	0.08	0.13	0.18
20:4n-6	0.08	0.13	0.18
22:2n-6	0.75	0.72	0.69
20:5n-3	0.28	0.34	0.40
22:6n-3	1.39	1.40	1.41

a The premix provided the following per kg of diet: Fe (as ferrous sulfate) 4.5 g/kg; Cu (as copper sulfate) 1.0 g/kg; Zn (as zinc sulfate) 6.0 g/kg; Mn (as manganese sulfate 3.0 g/kg; Co (as cobalt sulfate) 0.02 g/kg, Se 0.02 g/kg; I 0.04 g/kg; VA 250000 IU/kg; VD 30000 IU/kg; VE 25000 IU/kg.

### Carcass quality analysis

The carcass quality was measured during carcass segmentation. The 12th-rib eye muscle area (EMA) was measured by a planimeter after drawing the cross-sectional figure of the rib eye muscle with sulfuric acid paper. Rib fat thickness (RFT), back fat thickness (BFT), and abdominal fat thickness (AFT) were directly measured with vernier calipers (at the level of the 12th rib). RFT was measured at 110 mm, BFT was measured at 40 mm, and AFT was measured at 127 mm from the spinal column. Additionally, dressing percentage (DP) was calculated as (HCW/LBW) × 100.

### Meat quality analysis

#### Meat edible quality and nutritional component analysis

Following a 24-h postmortem phase, standard procedures were used to assess the edible quality of the meat (the ultimate pH value, texture, tenderness, and WHC). Furthermore, the initial and final pH values of the meat sample were determined by the insertion of a portable pH meter built-in temperature compensator at 2 cm depth in meat samples. We calibrated the pH meter using pH 4.0 and 6.86. For color, the values of b^*^ (yellowness), a^*^ (redness), and L^*^ (lightness) were determined on meat samples using a Minolta-ADCI machine. It was fitted with a standard xenon lamp within the close aperture of 8 mm set to Illuminant D65 with the observer angle of 2°. In addition, a Warner-Bratzler machine was used to measure the shear force (SF) of samples (1 × 1 × 2 cm^3^) parallel to the direction of muscle fibers). Then, a TPA analyzer (CT3, Brookfield) was used to determine the hardness, elasticity, viscosity, adhesion, cohesion, and chewiness of samples (1 × 1 × 1 cm^3^) parallel to the direction of muscle fibers. Furthermore, the cooked meat percentage was calculated by dividing the post-cook weight of the samples by the pre-cook weight. The water loss percentage was estimated as cooking loss from the original weight of the meat samples. All samples were individually vacuum-packed and then assigned to the same cooking batch, which was cooked using a Thermostatic Water Bath machine of 85 °C for 30 min. Similarly, thaw loss and drip loss were determined using the same cooking loss procedure. We hung meat samples in a refrigerator (4 °C) for 24 h to calculate drip loss. However, for thaw loss, the meat samples were unfrozen (average weight of 25 g) for 12 h in a refrigerator (4 °C).

Subsequently, after 48 h of postmortem analysis, crude protein, the quantity of moisture, total ash in the LL, and crude fat were determined using the standard AOAC procedures (16). The moisture content of meat samples was tested by thoroughly drying them in an oven at 105 ± 1 °C. The Kjeldahl technique and Soxhlet extraction method were used to determine the crude protein content and the crude fat level, respectively. Meat samples were burned in a crucible at 550 ± 5 °C for 4 h to determine the total ash concentration.

Triplicate technical replicates per sample were tested and the average value of the three measurements was used in subsequent analyses.

#### Analysis of the amino and fatty acid composition of meat

For AA analysis, 60 mg of meat samples were mixed with 0.5 ml of a mixture (methanol:acetonitrile:water = 2:2:1, v/v), ground in liquid nitrogen, and vortexed and sonicated for 60 s and 30 min, respectively (repeat twice). The mixture was then lyophilized and kept at −80 °C after being precipitated for 1 h at −20 °C and centrifuged for 20 min at 14,000 rcf and 4 °C. The samples were separated using Agilent's UHPLC (1290 Infinity). The mobile phase system consists of 25 mM ammonium formate and 0.08% FA in water comprised the A phase, whereas the B phase was prepared using 0.1% FA acetonitrile. Furthermore, the samples were placed in an autosampler at 4 °C with a column temperature of 40 °C, a flow rate of 250 μl/min, and a 1 μl of injection volume. Furthermore, the liquid phase gradient was set as follows: 0–12 min, B phase changed linearly from 90 to 70%. Then, the B phase was linearly decreased to 50% in 6 min; in 18–25 min, the B phase changed linearly from 50 to 40%, with a 5-min re-equilibration interval; and finally, the B phase was linearly increased to 90% in 0.1 min and sustained for 6.9 min. A quality control (QC) sample was set when the same number of experimental samples were separated every time in the sample queue to detect and evaluate the stability and repeatability of the system. For quantitative data collecting from MS, the multiple reaction monitoring (MRM) procedure was employed.

We poured 50 mg of meat samples with 1 ml of chloroform–methanol solution into a 2-ml glass centrifuge tube, sonicated at 30 min for FA analysis. The supernatant was methyl esterified in water for 30 min with 2 ml of a 1% sulfuric acid–methanol solution. Notably, the mixture was extracted with 1 ml of n-hexane, washed with 5 ml of pure water, and 25 μl of methyl salicylate (internal standard). The supernatant (500 μl) was collected and used for GC-MS detection with a split ratio of 10:1. The separation was carried out on a capillary column (30 m × 0.25 mm × 0.25 μm, DB-WAX) in GC (Agilent). The programmed temperature was set as follows: 40 °C for 5 min and then 220 °C at a rate of 10 °C/minute for 5 min. Helium was used as the carrier gas, with a flow rate of 1.0 ml/min, and QC was added by the same method and time interval as mentioned above. Inlet, ion source, and transfer line temperatures were 280 °C, 230 °C, and 250 °C, respectively. The MS was set to single ion monitoring (SIM) and operated in the Electron Impact Ionization (EI) source with electron energy set at 70 eV.

To collect qualitative and quantitative data on metabolites, MultiQuant and MSD ChemStation software were used to perform retention-time corrections, peak detection, and chromatographic alignment for AAs and FAs, respectively.

#### The analysis of meat's volatile flavor compounds

The meat samples' VOCs were detected using a GC-IMS system (Flavorspec, G.A.S. Instrument, Germany) with a SE-54 capillary column (15 m × 0.53 mm × 1 μm, Restek, United States). A 20 ml headspace vial was filled with a 2 g meat sample thawed in the refrigerator at 4 °C for 12 h, further preheated for 15 min at 80 °C. Following the incubation period, 500 μl of headspace gas was pumped into the injector at 85 °C. The temperature of the column was fixed at 60 °C, whereas the temperature of the drift tube was kept at 45 °C with carrier gas nitrogen (99.999% purity) at a flow rate of 150 ml/min (constant flow), and the GC column flow rate was set to 2 ml/min for 2 min. The flow was increased to 20 ml/min for 8 min, further to 100 ml/min for 15 min before being halted. The VOCs were determined by comparing the standard's retention index (RI) and drift time (Dt) in the NIST and IMS databases. The proportion of each component in meat samples was estimated using a normalized quantitative analytical approach of the peak volume for each component, respectively.

### Meat metabolomics profiling

Meat samples were processed with a pre-cooled mixture composed of methanol (2): acetonitrile (2): water (1) v/v, were ground in liquid nitrogen, vortexed, and sonicated for 60 s and 30 min, respectively. Additionally, annealing was performed at −20 °C for 10 min followed by 20 min of centrifugation at 14,000 rcf. Moreover, the obtained supernatant was evacuated and redissolved by centrifugation using the same parameters with 100 μl of the mixture (acetonitrile:water = 1:1, v/v) for UHPLC-QTOF-MS (1,290 Infinity, Agilent) analysis. Throughout the analysis, the meat samples were located at 4 °C in an autosampler having an injection volume of 2 μl with a 0.5 ml/min flow rate and a column temperature of 25 °C, respectively. The mobile phase system consisting of 25 mM ammonium acetate and ammonium in water comprised the A phase while the B phase was prepared using acetonitrile. The liquid phase gradient appropriate for observing the separation of compounds between two phases was as follows: for 0.5 min, the B phase was 95% B; from 0.5 to 7 min, a linear B phase shift was observed from 95 to 65%. Furthermore, linear lowering of the B phase was achieved from 40% during 1 min and lasted about 1 min, and after that, the B phase was increased by about 95% for 0.1 min and lasted for about 2.9 min. To prevent the effects of signal fluctuations, the samples were evaluated at random and in real-time. To monitor and assess the system's stability and the dependability of experimental data, QC samples were added to the sample queue.

The first- and second-order spectrums of the samples were collected using an MS (AB Triple TOF 6600). The Electron Spray Ionization (ESI) source conditions were set as follows: both Ion Source Gas2(Gas2) and Ion Source Gas1(Gas1): 60, source temperature: 600 °C, CUR: 30, ISVF: ± 5500, product ion, and TOF MS scan m/z range were set as 25–1000 and 60–1000 Da, respectively, product ion and TOF MS scan accumulation time were set as 0.05 and 0.20 s/spectra, respectively. The second-order spectrum was obtained using information-dependent acquisition (IDA) and the high sensitivity mode. XCMS software was used to do the alignment, retention-time adjustments, and peak detection. Furthermore, SIMCA software (version 14.0) was used to conduct multivariate statistical analysis, i.e., discriminant analysis of partial least squares (PLS-DA), principal component analysis (PCA), and discriminant analysis of orthogonal partial least squares (OPLS-DA), and R package was used to generate the heat maps. Differential metabolites (DMs) were defined as those having a VIP value of more than 2 and a *P*-value < 0.05. By comparing the mean value between HS and MS, HS and LS, and MS and LS, the fold-change value of each metabolite was calculated. The database from the Kyoto Encyclopedia of Genes and Genomes (KEGG, www.genome.jp/kegg) was assessed for functional annotation and enrichment analysis of metabolic pathways.

### The composition of the rumen microbiota and the characteristics of the rumen fermentation analysis

#### Rumen fermentation characteristics

The pH value of the rumen fluid was immediately evaluated using a portable pH meter after it was collected in a sterile centrifuge tube of 50 ml. GC (NX 2030, Shimadzu) was used to determine the concentrations of volatile fatty acids (VFAs), while ammonia-N concentration was evaluated using the phenol–hypochlorite assay (17).

#### DNA extraction, amplification of 16S RDNA, and MiSeq sequencing

Eventually, The CTAB/SDS technique was used to extract complete genomic DNA from rumen fluid samples. The obtained DNA concentration and purity were evaluated on 1% agarose gels. The specific primer containing the barcode was used to amplify the V3–V4 regions of 16S rRNA genes. All PCR reactions were carried out in a 30 μl mixture having 0.2 M of forward and reverse primers, 15 μl of New England Biolabs' Phusion® High-Fidelity PCR Master Mix, and 10 ng of template DNA. The thermal cycle program of PCR was set as follows: denaturation for 1 min at 98 °C; 10 s of 30 cycles at 98 °C, annealing for 30 s at 50 °C, elongation for 60 s at 72 °C; and finally, extension at 72 °C for 5 min. Moreover, the obtained PCR products were loaded to 2% agarose gel with the same volume of 1X loading buffer (contained SYB green). The DNA was further purified and quantified using AxyPrep DNA Gel Extraction Kit (AXYGEN, USA), QuantiFluor™-ST (Promega, USA), applying the reported protocol. A sequencing library was generated using NEB Next® Ultra™DNA Library Prep Kit for Illumina (NEB, USA) and library quality was assessed on Qubit@ 2.0 Fluorometer (Thermo Scientific) and Agilent Bioanalyzer 2100 system. Finally, the library was sequenced on an Illumina Miseq/HiSeq2500 platform and 250bp/300bp paired-end reads were generated.

FLASH was used to combine paired-end reads from the original DNA fragments and assign each sample a unique barcode. The UPARSE-OTUref and UPARSE-OTU packages of UPARSE software were used to analyze the sequences. The diversity of Alpha (within samples) and beta (among samples) was analyzed using in-house Perl scripts. The OTUs were assigned to sequences with ≥ 97% similarity and annotated with taxonomic information through the RDP classifier. Both weighted and unweighted unifrac distances were calculated by QIIME. For principal coordinate analysis (PCoA), we employed weighted unifrac. The difference in the abundance of individual taxonomies among distinct groups was confirmed using the STAMP program. The quantitative analysis of biomarkers within the different groups was performed with LEfSe. QIIME was used to calculate the Chao1, Simpson, and other alpha indexes. The Bray-Curtis dissimilarity distance matrices were used to accomplish Anosim and Adonis.

### Statistical analysis

A general linear model (GLM, SPSS 22.0) was used to evaluate the effects of dietary treatment as the fixed factor on the traits (carcass quality, meat quality, muscle metabolism, rumen fermentation characteristics, and rumen microbiota composition) of Black Tibetan sheep. All data were analyzed using one-way ANOVA to obtain the mean and the standard error of the mean (SEM). A value of *P* < 0.05 was used to assess the significance. The relationship between meat quality, muscle metabolism, and rumen bacteria of Black Tibetan sheep under different dietary treatments was analyzed by Pearson's correlation coefficient. A *P*-value of < 0.05 and |r| > 0.50 were considered to have a significant correlation.

## Results

### Carcass quality

The carcass quality of Black Tibetan sheep fed on different energy level diets is shown in Table 2. There was no difference in the EMA, RFT, BFT, and AFT of sheep among the three feed groups (LS, MS, and HS). However, the LBW, HCW, and DP of sheep were higher (*P* < 0.05) in HS compared to MS, while no significant difference was found between MS and LS. Additionally, the HS group's HCW was greater than the LS group's (*P* < 0.05). These findings indicated that HS gives the best carcass quality.

**Table 2:** Effect of different energy level diets on the carcass quality of Tibetan sheep.

**Items**	**Groups**			**SEM**	***P*-value**
	**HS**	**MS**	**LS**		
Eye muscle area (cm^2^)	22.97	18.52	18.24	2.93	0.14
Rib fat thickness (cm)	3.38	3.67	3.50	0.36	0.73
Back fat thickness(cm)	5.81	5.02	4.86	0.79	0.24
Abdominal fat thickness (cm)	5.49	5.28	4.68	0.53	0.15
Live body weight (kg)	33.93^a^	28.80^b^	30.87^a^^b^	1.29	0.16
Hot carcass weight (kg)	17.12^a^	12.63^b^	13.82^b^	0.88	0.06
Dressing percentage (%)	50.52^a^	43.89^b^	44.73^a^^b^	2.57	0.08

a,b means within a row with different subscripts differ when P-value < 0.05.

### Meat quality

#### Edible quality and nutritional components of meat

Several meat quality characteristics, such as viscosity, ultimate pH, elasticity, color, shear force, and cohesiveness, were unaffected by dietary energy levels (Table 3). Consequently, the initial pH, thaw loss, drip loss, cooking loss, hardness, adhesion, and chewiness of the LL were considerably lower (*P* < 0.05) while the cooked meat percentage of the LL was considerably higher (*P* < 0.05) in HS than in MS and LS. Notably, except for the drip loss, which was lower in MS compared to LS (*P* < 0.01), there was no difference in the rest of the parameters between MS and LS. The dietary energy levels significantly affected the fat content of the LL. MS had a reduced fat level (*P* < 0.01) compared to HS. As a result, the HS group had better meat edible quality, including WHC and texture, as well as better meat nutritional quality, including fat content.

**Table 3:** Effect of different energy level diets on the edible quality and nutritional components of the longissimus lumborum of Tibetan sheep.

**Items**	**Groups**			**SEM**	***P*-value**
	**HS**	**MS**	**LS**		
The edible quality					
Initial pH (0 h)	6.05^b^	6.43^a^	6.52^a^	0.06	<0.01
Ultimate pH (24 h)	5.77	5.82	5.82	0.04	0.16
Color (45 min)					
L^*^	26.00	27.28	25.72	1.10	0.81
a^*^	9.67	7.84	9.73	1.87	0.98
b^*^	5.81	5.46	6.77	1.03	0.36
Shear force (N)	11.01	10.22	9.01	1.09	0.07
Thaw loss (%)	7.24^b^	10.19^a^	10.44^a^	0.39	<0.01
Drip loss (%)	3.94^c^	5.14^b^	7.09^a^	0.49	<0.01
Cooking loss (%)	17.39^b^	25.99^a^	24.05^a^	2.55	0.02
Cooked meat percentage (%)	76.72^a^	69.57^b^	69.89^b^	2.70	0.02
Hardness (g)	758.33^b^	2120.44^a^	1912.22^a^	145.04	<0.01
Elasticity (mm)	1.54	1.62	1.55	0.08	0.86
Viscosity (mJ)	0.16	0.18	0.12	0.03	0.28
Adhesion (g)	263.56^b^	545.33^a^	596.56^a^	56.83	<0.01
Cohesion	0.35	0.29	0.30	0.04	0.18
Chewiness (mJ)	5.14^b^	8.52^a^	7.71^a^	0.86	0.01
The nutritional components (%)					
Moisture	74.58	75.36	75.08	0.57	0.39
Ash	1.00	1.01	0.96	0.03	0.21
Fat	3.77^a^	2.73^b^	3.23^a^^b^	0.26	0.10
Protein	21.22	21.30	21.04	0.52	0.74

a,b,c means within a row with different subscripts differ when P-value < 0.05.

#### Meat volatile flavor compound analysis

**Figure 1:**
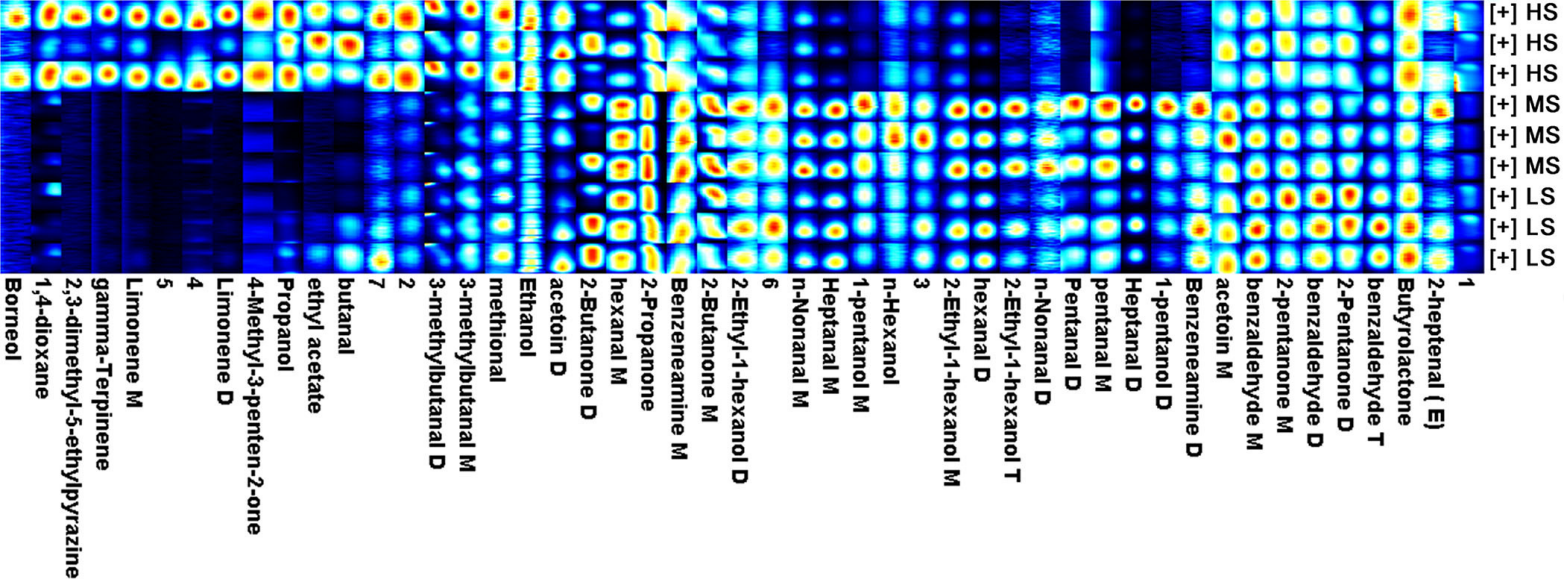
The fingerprint of volatile flavor compounds obtained from the longissimus lumborum of Tibetan sheep under different energy level diets. M: monomer, D: dimer, T: polymer. 1–7 means unidentified compounds.

Gas chromatography-IMS analysis revealed a total of 49 VOCs, including 16 aldehydes, 11 alcohols, 2 esters, 6 ketones, 2 heterocycles, 2 amines, 3 terpenoids, and 7 unknown compounds. As shown in Figure 1, ethyl acetate, propanol, 4-methyl-3-penten-2-one, 2,3-dimethyl-5-ethylpyrazine, limonene D, limonene M, 1,4-dioxane, borneol, and gamma-terpinene were only detected in the HS group, but benzeneamine D, 1-pentanol D, heptanal D, pentanal M, pentanal D, and n-nonanal D were absent in this group. Terpenoids and heterocycles were only present in the HS group. As a result, the HS group was observed to have more VOCs than the MS and LS groups. The relative proportion of VOCs in each group is shown in Table 4. The MS group had the largest proportion of aldehydes, followed by the LS group, which had a medium rate, and the HS group had the lowest percentage (*P* < 0.01). Additionally, the HS group possessed a high alcohol proportion compared to the MS group (*P* < 0.01) while a high proportion (*P* < 0.05) of ketone was calculated in the LS group compared to the HS group. Notably, HS was having a lower concentration of hexanal M and benzaldehyde M compared with MS and LS (*P* < 0.01). However, the concentration of benzaldehyde D in HS was lower than that in LS (*P* < 0.05). Similarly, HS had a lower benzaldehyde T than LS (*P* < 0.05), whereas MS was in the median (*P* < 0.05). Moreover, the results of heptanal M, hexanal D, and n-nonanal M were the same as the result of total aldehyde content. These findings show that dietary energy levels affected the flavor of Black Tibetan sheep mutton.

**Table 4:** Effect of different energy level diets on the volatile flavor compounds in the longissimus lumborum of Tibetan sheep (% of total volatile flavor compounds).

**Items**	**Groups**			**SEM**	***P*-value**
	**HS**	**MS**	**LS**		
hexanal M	1.64^b^	4.17^a^	3.48^a^	0.35	0.05
hexanal D	0.87^c^	6.19^a^	4.44^b^	0.32	0.06
Heptanal M	0.95^c^	4.45^a^	3.35^b^	0.18	0.05
n-Nonanal M	1.29^c^	2.94^a^	2.06^b^	0.13	0.22
benzaldehyde M	2.46^b^	3.22^a^	3.33^a^	0.20	<0.01
benzaldehyde D	0.46^b^	0.60^a^^b^	0.68^a^	0.07	0.01
benzaldehyde T	0.79^c^	1.10^b^	1.35^a^	0.08	<0.01
aldehydes	12.75^c^	30.56^a^	25.32^b^	1.14	0.04
alcohols	50.27^a^	41.17^b^	45.80^a^^b^	2.12	0.25
ketones	20.06^b^	24.34^a^^b^	25.75^a^	1.84	0.02
esters	0.49	0.53	0.52	0.06	0.53
terpenoids	2.19	–	–	–	–
heterocycles	6.03	–	–	–	–

a,b,c means within a row with different subscripts differ when P-value < 0.05. M: monomer, D, dimer; T, polymer. –means represents undetected compound.

#### The composition of AAs and FAs

The dietary energy level only slightly affected the AA and FA levels in Black Tibetan sheep mutton (Tables 5, 6). There was no difference in the concentration of EAAs, NEAAs, and TAAs in the LL among the three groups. However, the concentration of glycine, serine, asparagine, and glutamine were greater in MS than in LS (*P* < 0.05). Furthermore, MS had a higher choline level than HS and LS (*P* < 0.05), while HS had a higher taurine level than MS (*P* < 0.05). There was no difference in the SFA, MUFA, and PUFA concentrations between the three groups according to the FA profile.

**Table 5:** Effect of different energy level diets on the amino acid composition in the longissimus lumborum of Tibetan sheep (mg/100 g tissue).

**Items**	**Groups**			**SEM**	***P*-value**
	**HS**	**MS**	**LS**		
Glycine	32.32^a^^b^	53.05^a^	31.42^b^	8.55	0.94
Serine	5.54^a^^b^	6.81^a^	4.30^b^	0.89	0.32
Asparagine	4.39^a^^b^	6.52^a^	3.63^b^	1.07	0.62
Glutamine	121.26^a^^b^	154.41^a^	92.50^b^	21.54	0.35
Taurine	101.47^a^	45.13^b^	57.83^a^^b^	21.64	0.13
Choline	2.21^b^	3.55^a^	2.62^b^	0.32	0.50
EAAs	34.42	34.89	30.95	4.72	0.46
NEAAs	478.20	490.01	396.26	82.66	0.34
TAAs	512.62	524.90	427.21	86.57	0.34

a,b means within a row with different subscripts differ when P-value < 0.05. Only amino acids with a significant difference in at least one of the comparisons were presented. EAAs, essential amino acids; NEAAs: non-essential amino acids; TAAs, total amino acids.

**Table 6:** Effect of different energy level diets on the fatty acid composition in the longissimus lumborum of Tibetan sheep (mg/100 g tissue).

**Items**	**Groups**			**SEM**	***P*-value**
	**HS**	**MS**	**LS**		
∑SFA	301.02	306.82	483.16	107.09	0.13
6:0	0.00	0.00	0.01	0.00	0.65
10:0	0.10	0.11	0.23	0.07	0.09
11:0	0.00	0.00	0.01	0.00	0.05
12:0	0.39	0.43	0.79	0.22	0.10
13:0	0.02	0.03	0.06	0.02	0.10
14:0	13.01	11.92	24.47	7.67	0.18
15:0	1.22	1.30	2.68	0.74	0.09
16:0	156.01	162.51	243.58	54.25	0.14
17:0	4.90	5.16	9.49	2.28	0.09
18:0	123.74	123.74	199.51	41.95	0.12
20:0	0.87	0.83	1.48	0.35	0.13
21:0	0.36	0.38	0.35	0.03	0.70
22:0	0.15	0.14	0.22	0.05	0.20
23:0	0.09	0.09	0.10	0.01	0.44
24:0	0.16	0.18	0.20	0.02	0.10
∑MUFA	283.92	288.99	400.68	84.11	0.19
c9-14:1	0.44	0.37	0.64	0.22	0.39
c10-15:1	0.35	0.34	0.63	0.16	0.12
c9-16:1	13.00	12.17	19.69	5.67	0.27
c10-17:1	4.02	4.25	6.80	1.29	0.07
c9-18:1	263.03	269.05	368.89	76.21	0.19
c11-20:1	2.25	2.06	2.86	0.51	0.28
c13-22:1	0.83	0.75	1.17	0.32	0.32
∑PUFA	85.84	79.72	86.36	6.56	0.94
∑n-3	27.46	27.09	27.37	0.97	0.92
18:3n-3	2.65	2.42	3.40	0.48	0.19
20:3n-3	18.78^a^	18.88^a^	16.89^b^	0.59	0.03
20:5n-3	1.44^a^^b^	1.29^b^	1.89^a^	0.19	0.11
22:5n-3	3.91	3.95	4.47	0.34	0.13
22:6n-3	0.68	0.55	0.72	0.10	0.71
∑n-6	58.37	52.63	58.99	5.62	0.92
18:2n-6	52.01	46.67	53.89	5.42	0.75
18:3n-6	0.62	0.50	0.53	0.08	0.29
20:3n-6	1.87	1.68	1.89	0.13	0.88
20:4n-6	0.28	0.27	0.25	0.02	0.15
22:2n-6	0.04	0.04	0.03	0.02	0.67
22:4n-6	2.75^a^	2.74^a^	1.89^b^	0.29	0.03
22:5n-6	0.80^a^	0.74^a^	0.51^b^	0.06	<0.01

a,b means within a row with different subscripts differ when P-value < 0.05. c: cis. ∑SFA: sum of saturated fatty acids (6:0, 10:0, 11:0, 12:0, 13:0, 14:0, 15:0, 16:0, 17:0, 18:0, 20:0, 21:0, 22:0, 23:0, 24:0); ∑MUFA: sum of monounsaturated fatty acids (c9-14:1, c10-15:1, c9-16:1, c10-17:1, c9-18:1, c11-20:1, c13-22:1); ∑PUFA: sum of polyunsaturated fatty acids (∑n-3 + ∑n-6); ∑n-3: sum of omega-3 polyunsaturated fatty acids (18:3n-3, 20:3n-3, 20:5n-3, 22:5n-3, 22:6n-3); ∑n-6: sum of omega-6 polyunsaturated fatty acids (18:2n-6, 18:3n-6, 20:3n-6, 20:4n-6, 22:2n-6, 22:4n-6, 22:5n-6).

### Metabolomics analysis of the muscle

**Figure 2:**
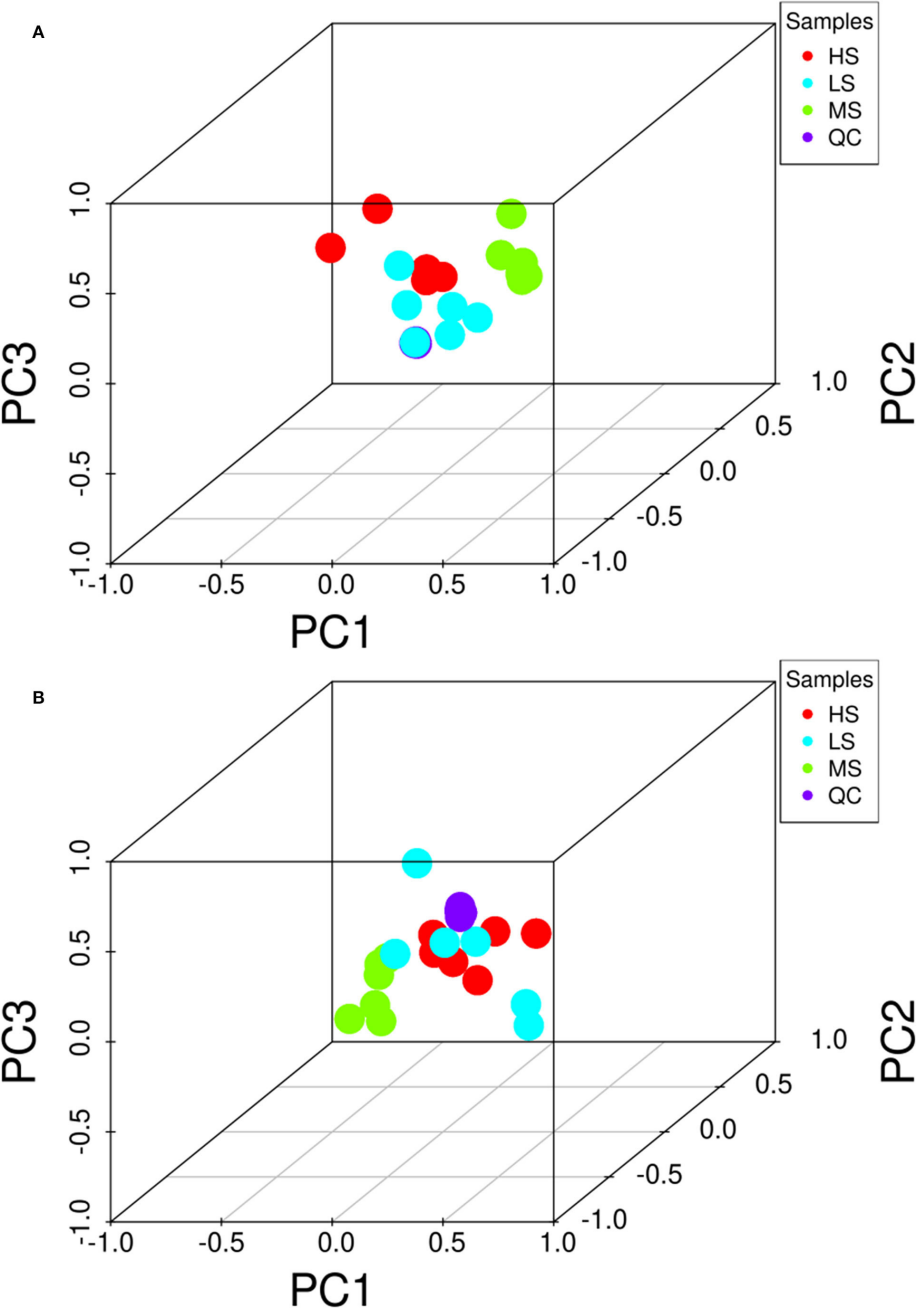
3D PCA score of the overall samples in the positive **(A)** and negative **(B)** ion detection mode. The HS group is marked as red, the MS group is marked as green, the LS group is marked as blue, and the QC samples are indicated with purple. PC1 represents principal component 1, PC2 represents principal component 2, and PC3 represents principal component 3.

**Figure 3:**
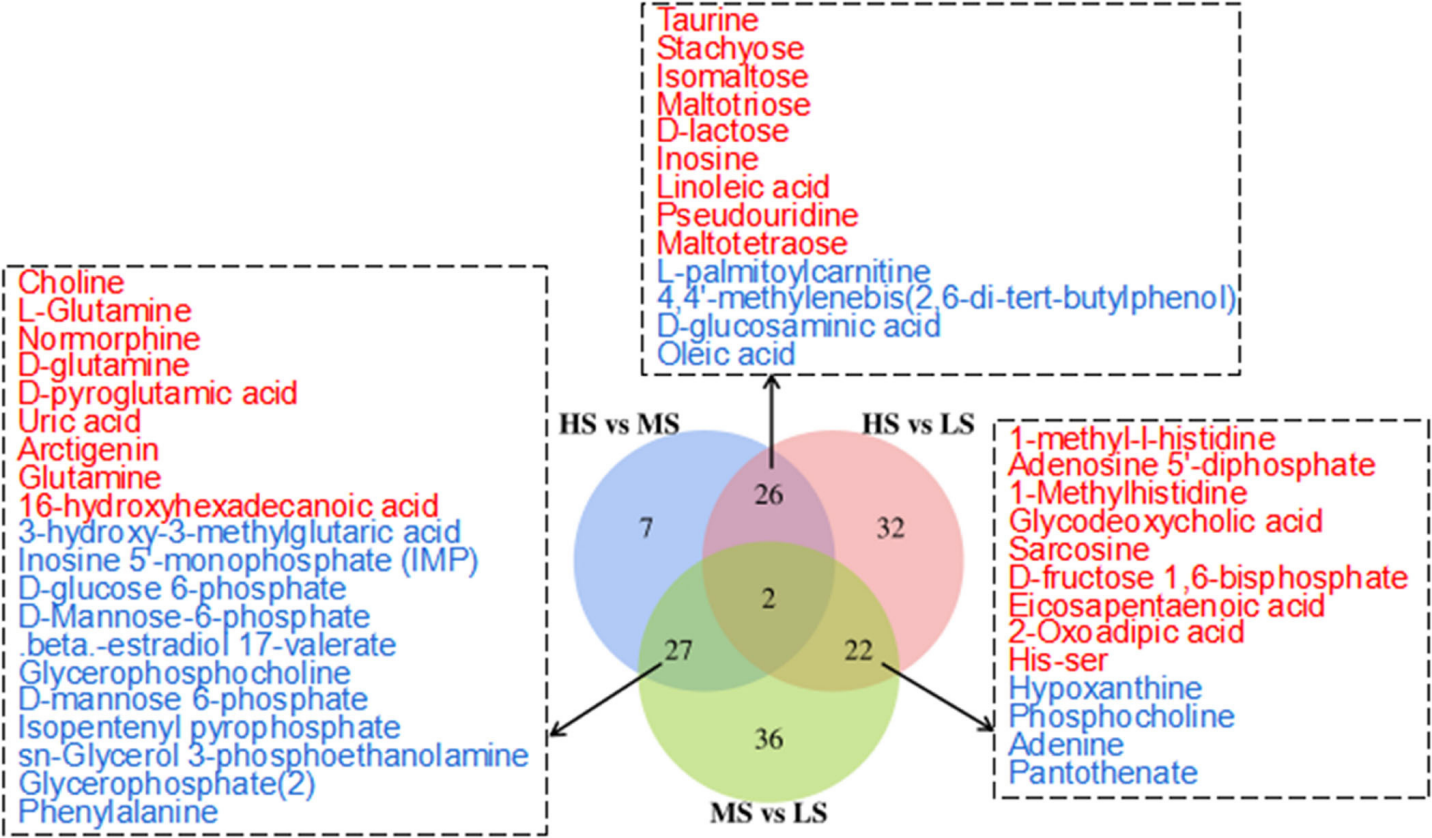
Venn diagram illustrates the overlap of differential metabolites connected with the KEGG metabolic pathways among the three comparisons (HS vs. MS; HS vs. LS; MS vs. LS) in the longissimus lumborum of Tibetan sheep. The color red and blue represent the upregulation and downregulation of metabolites, respectively.

Figure 2 shows the 3D PCA score plots of the negative and positive ion modes for the three groups and controls. The 3D PCA score plots demonstrated a marked distinction between the three groups. The dissections among the three groups were validated using PLS-DA (Supplementary Figure S1) and OPLS-DA (Supplementary Figure S2). OPLS-DA analysis revealed a separation and aggregation within and between HS and MS group in positive (R^2^X = 0.329, R^2^Y = 0.990, Q^2^ = 0.843) and negative (R^2^X = 0.585, R^2^Y = 0.999, Q^2^ = 0.554) modes. Furthermore, OPLS-DA analysis results in the separation and aggregation found within and among the HS and LS groups in terms of positive (R^2^X = 0.377, R^2^Y = 0.992, Q^2^ = 0.863) and negative (R^2^X = 0.509, R^2^Y = 0.993, Q^2^ = 0.741) modes. Additionally, the OPLS-DA analysis also resulted in the difference in separation and aggregation as positive (R^2^X = 0.544, R^2^Y = 0.995, Q^2^ = 0.760) and negative (R^2^X = 0.298, R^2^Y = 0.969, Q^2^ = 0.521) modes within and between MS and LS groups, demonstrating that dietary energy levels influence the LL of Black Tibetan sheep metabolic pathways. Analysis of differential metabolites (DMs) identified 62 DMs (31 and 31 in the negative and positive mode, respectively) between HS and MS, 82 DMs (40 and 42 in the negative and positive mode, respectively) between HS and LS, and 87 DMs (55 and 32 in the negative and positive mode, respectively) between MS and LS. Compared with LS and MS, 26 metabolite levels were significantly different in HS (17 DMs were lower, whereas 9 DMs were significantly higher). Also, compared with the other groups, 27 metabolite levels were different in MS (12 DMs were significantly high, whereas 15 DMs were significantly low). Finally, the levels of 22 metabolites in the LS group were substantially different from the other groups (13 DMs were significantly high, whereas 9 DMs were low). A total of 46 DMs associated with the metabolic pathways, including AAs, FAs, organic acids, nucleotides, and carbohydrates, were assessed using KEGG analyses (Figure 3).

**Figure 4:**
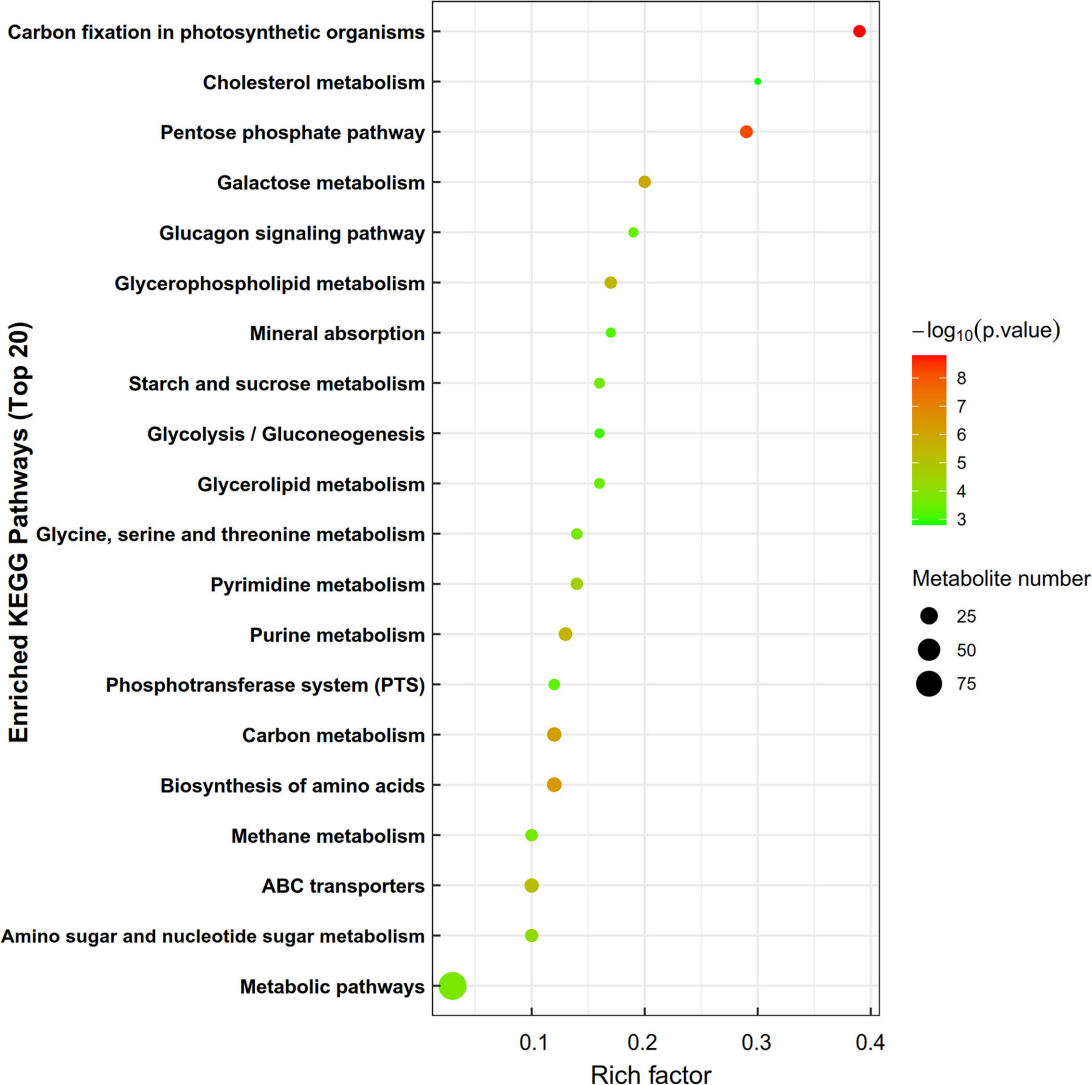
The enrichment of KEGG metabolic pathways of differential metabolites in the longissimus lumborum of Tibetan sheep. In the bubble diagram, each bubble represents a metabolic pathway. The larger the bubble is, the greater the impact factor is; the darker the bubble is, the more significant the degree of enrichment is.

Kyoto Encyclopedia of gene and genomes enrichment analysis revealed that the level of dietary energy mainly affected transmembrane transport and the metabolism of lipids, amino acids, nucleotides, and carbohydrates (Figure 4). Differential abundance analysis (Table 7) revealed that compared to MS, 8 and 7 metabolic pathways were upregulated and downregulated in HS, respectively (> 0.5 DA score; *P* < 0.05). The key upregulated pathways included carbohydrate digestion and absorption, glycerophospholipid metabolism, ether lipid metabolism, galactose metabolism, phosphotransferase system (PTS), and starch and sucrose metabolic pathways in HS. The levels of several metabolites, including glycerophosphocholine, D-glucose 6-phosphate, glycerophosphate (2), maltotriose, sn-glycerol 3-phosphoethanolamine, isomaltose, D-lactose, stachyose, phenylalanine, D-mannose 6-phosphate, D-glucose 6-phosphate, and D-glucosaminic acid, were higher in the HS group. In the HS group, D-glutamine and D-glutamate metabolism pathway was suppressed, whereas glutamine, L-glutamine, choline, D-glutamine, D-pyroglutamic acid, and D-mannose-6-phosphate levels were all lower. Compared with LS, 4 metabolic pathways were upregulated, whereas 5 were downregulated in the HS group (> 0.5 DA score; *P* < 0.05). Galactose metabolism, carbohydrate digestion and absorption, and purine metabolism were the key upregulated pathways, whereas the AMPK signaling pathway was downregulated in HS. The key upregulated metabolites included maltotriose, D-lactose, stachyose, adenine, inosine, and hypoxanthine, whereas adenosine 5'-diphosphate and D-fructose 1,6-bisphosphate were the key downregulated metabolites in HS. Compared to LS, 12 metabolic pathways were upregulated and 15 were downregulated in MS (> 0.5 DA score; *P* < 0.05). The key upregulated pathways included D-glutamine and D-glutamate metabolism pathways, whereas the metabolic pathways of galactose, glycerolipid, ether lipid, starch and sucrose, and amino sugar and nucleotide sugar metabolism were all downregulated in MS. The key downregulated metabolites included D-pyroglutamic acid, glutamine, L-glutamine, D-glutamine, and DL-2-Phosphoglycerate in MS. Alpha-D-galactose 1-phosphate, dihydroxyacetone phosphate, udp-n-acetylglucosamine, D-glucose 6-phosphate, sn-glycerol 3-phosphoethanolamine, D-mannose 6-phosphate, glycerophosphate (2), glycerophosphocholine, D-fructose-6-phosphate, D-glucosamine 6-phosphate, D-mannose-6-phosphate, alpha-D-glucose 1-phosphate metabolites were all upregulated in MS. In brief, compared with the other groups, carbohydrate metabolism was significantly upregulated in HS, whereas lipid metabolism was considerably downregulated in MS. More importantly, dietary energy levels affected both the PTS and AMPK signaling pathways in the LL of Black Tibetan sheep.

**Table 7:** Differential metabolites in the key metabolic pathways among the three comparisons (HS vs. MS; MS vs. LS; and MS vs. LS) in the longissimus lumborum of Tibetan sheep (accounting for the absolute value of differential abundance score ≥ 0.5).

**Metabolic Pathways**	**Metabolites**
	HS vs. MS
upregulation in the HS group	
Glycerophospholipid metabolism	sn-Glycerol 3-phosphoethanolamine, Glycerophosphate(2), Choline, Glycerophosphocholine
Carbohydrate digestion and absorption	Maltotriose, D-lactose, D-glucose 6-phosphate
Ether lipid metabolism	sn-Glycerol 3-phosphoethanolamine, Glycerophosphocholine
Starch and sucrose metabolism	Isomaltose, D-glucose 6-phosphate
Galactose metabolism	D-lactose, Stachyose
Phosphotransferase system (PTS)	D-lactose, D-mannose 6-phosphate, D-glucose 6-phosphate, D-glucosaminic acid, D-Mannose-6-phosphate
downregulation in the HS group	
D-Glutamine and D-glutamate metabolism	Glutamine, L-Glutamine, D-glutamine, D-pyroglutamic acid
	HS vs. LS
upregulation in the HS group	
Carbohydrate digestion and absorption	Maltotriose, D-lactose
Galactose metabolism	D-lactose, Stachyose
Purine metabolism	Adenine, Inosine, Hypoxanthine, Adenosine 5'-diphosphate
downregulation in the HS group	
AMPK signaling pathway	D-fructose 1,6-bisphosphate, Adenosine 5'-diphosphate
	MS vs. LS
upregulation in the MS group	
D-Glutamine and D-glutamate metabolism	Glutamine, D-pyroglutamic acid, L-Glutamine, D-glutamine
downregulation in the MS group	
Galactose metabolism	Dihydroxyacetone phosphate, alpha-D-Galactose 1-phosphate, alpha-D-Glucose 1-phosphate, D-Fructose-6-phosphate
Amino sugar and nucleotide sugar metabolism	Udp-n-acetylglucosamine, D-mannose 6-phosphate, D-glucosamine 6-phosphate, alpha-D-Galactose 1-phosphate, alpha-D-Glucose 1-phosphate, D-Mannose-6-phosphate
Glycerolipid metabolism	Dihydroxyacetone phosphate, Glycerophosphate(2), DL-2-Phosphoglycerate, alpha-D-Glucose 1-phosphate
Starch and sucrose metabolism	D-glucose 6-phosphate, alpha-D-Glucose 1-phosphate, D-Fructose-6-phosphate
Ether lipid metabolism	sn-Glycerol 3-phosphoethanolamine, Glycerophosphocholine

### Characteristics of the rumen fermentation and composition of the rumen microbiota analysis

#### Change in rumen fermentation characteristics

Table 8 shows the rumen fermentation parameters of Black Tibetan sheep fed various energy level diets. The ammonia-N concentration in the three groups was not significantly different. The pH of rumen fluid in HS, however, was lower (*P* < 0.01) than that in MS and LS. HS had considerably higher levels of 5 VFAs, including valerate, isovalerate, butyrate, propionate, and acetate compared to MS and LS (*P* < 0.05). Furthermore, LS had a higher acetate to propionate (A/P) ratio than HS and MS (*P* < 0.01).

**Table 8:** Effect of different energy level diets on rumen fermentation characteristics of Tibetan sheep.

**Items**	**Groups**			**SEM**	***P*-value**
	**HS**	**MS**	**LS**		
pH	5.71^b^	6.11^a^	6.20^a^	0.13	<0.01
Ammonia-N (mmol/L)	21.68	22.26	17.74	3.20	0.23
Acetate (mmol/L)	60.10^a^	48.36^b^	52.41^b^	3.10	0.04
Propionate (mmol/L)	23.42^a^	17.63^b^	17.07^b^	1.30	<0.01
Isobutyrate (mmol/L)	0.83	0.86	0.86	0.17	0.83
Butyrate (mmol/L)	18.37^a^	12.20^b^	15.99^a^^b^	2.29	0.35
Isovalerate (mmol/L)	3.65^a^	2.21^b^	1.95^b^	0.44	<0.01
Valerate (mmol/L)	1.35^a^	0.94^b^	0.89^b^	0.17	0.01
Total VFAs (mmol/L)	107.71^a^	82.20^b^	89.18^b^	4.90	<0.01
A/P	2.57^b^	2.80^b^	3.06^a^	0.13	<0.01

a,b means within a row with different subscripts differ when P-value < 0.05. VFAs: volatile fatty acids; A: acetate; P: propionate.

#### Change in rumen microbiota composition

**Figure 5:**
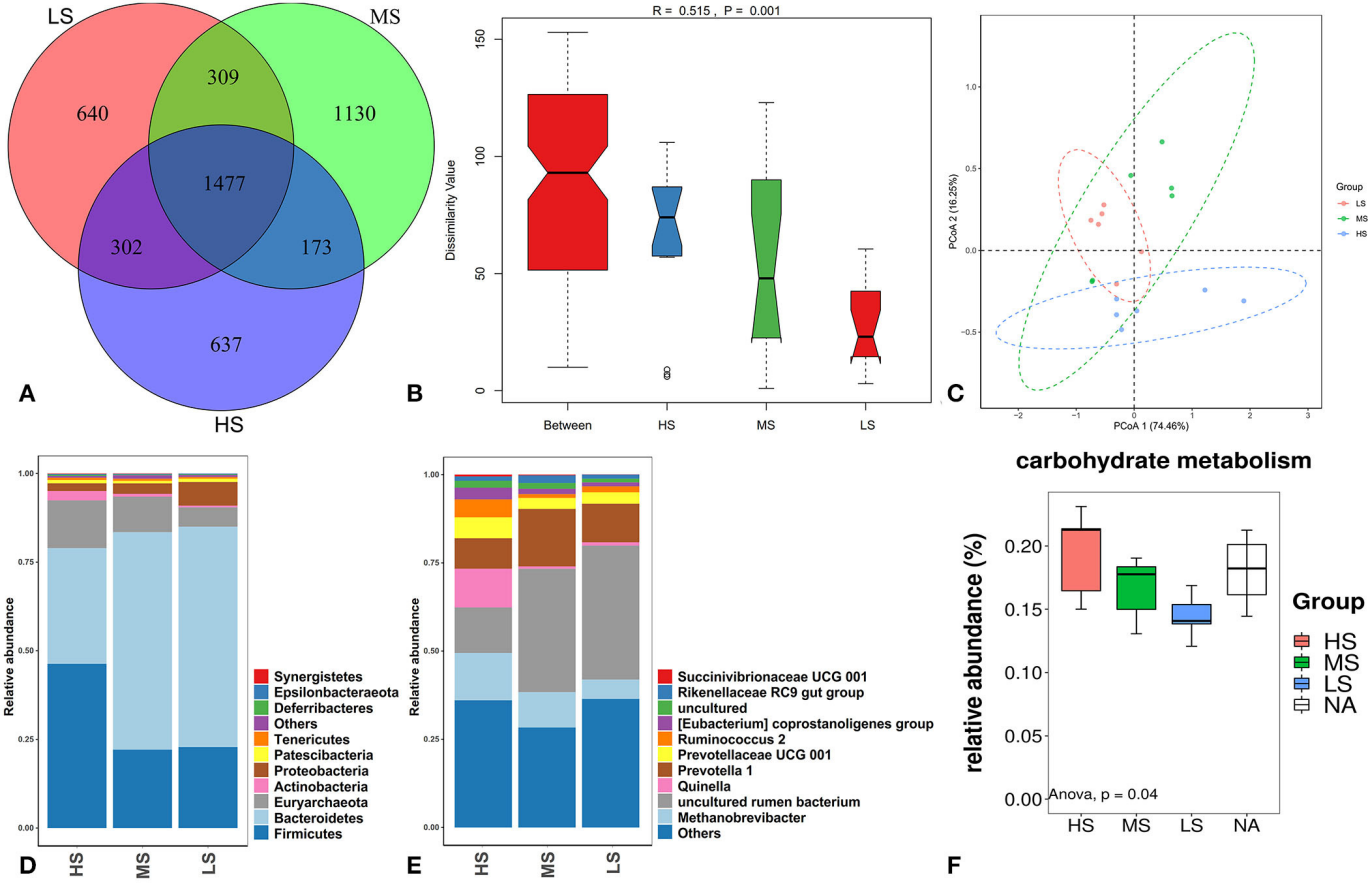
OTUs Venn diagram of the overlap of rumen microbiota among the three groups **(A)**. Anosim analysis **(B)** and PCoA plot **(C)** of the overall samples of rumen microbiota. Relative abundance of bacteria community proportion at the phylum **(D)** and genus **(E)** levels among the three groups. Boxplot of the prediction of the most important diff rumen function **(F)**.

A total of 1,477 OTUs were identified across the three groups. Of these, 637, 1,130, and 640 OTUs were identified in HS, MS, and LS, respectively (Figure 5A). Shannon, Simpson, Ace, and Chao1 bacterial diversity indexes were similar among the three groups. Anosim analysis (Figure 5B) and PCoA plot (Figure 5C) revealed a significant difference and good separation among the three groups, indicating a change in the rumen microbiota of Black Tibetan sheep under different dietary conditions. Based on relative abundance ≥ 0.01%, 102 bacteria genus in 24 phyla were identified. The top 10 most abundant phylum and genera are shown in Figures 5D,E, respectively. Four and six differently abundant phyla and genera among the three groups are shown in Table 9. At the phylum level, compared with MS and LS, *Actinobacteria* and *Firmicutes* were more abundant in HS, contrarily, *Bacteroidetes* was less abundant in HS. Moreover, *Synergistetes* was less abundant in LS than that in the others. At the genus level, the abundance of *Quinella, Ruminococcus 2, (Eubacterium) coprostanoligenes*, and *Succinivibrionaceae UCG-001* were greater in HS than those in MS and LS. However, the abundance of uncultured rumen bacterium was lower in HS than that in MS and LS. The abundance of *Rikenellaceae RC9 gut* was higher in MS than that in HS and LS. Further function analyses revealed that the different abundant rumen microbiota regulated carbohydrate metabolism (Figure 5F).

**Table 9:** The main differential rumen bacteria at the phylum and genus levels among the three groups (accounting for the relative abundance in top 10).

**Items**	**Groups**			**SEM**	***P*-value**
	**HS**	**MS**	**LS**		
Phylum level (%)					
Actinobacteria	2.65^a^	0.79^b^	0.47^b^	0.54	<0.01
Bacteroidetes	32.66^b^	61.39^a^	62.18^a^	4.64	<0.01
Firmicutes	46.32^a^	22.08^b^	22.82^b^	3.42	<0.01
Synergistetes	0.15^a^	0.12^a^	0.03^b^	0.04	<0.01
Genus level (%)					
Uncultured rumen bacterium	12.91^b^	34.97^a^	37.99^a^	8.17	<0.01
Quinella	10.98^a^	0.66^b^	0.94^b^	0.49	<0.01
Ruminococcus 2	5.08^a^	1.08^b^	1.67^b^	1.37	0.04
[Eubacterium] coprostanoligenes group	3.30^a^	1.51^b^	1.11^b^	0.59	<0.01
Rikenellaceae RC9 gut group	1.23^b^	2.16^a^	1.16^b^	0.35	0.88
Succinivibrionaceae UCG-001	0.52^a^	0.22^b^	0.00^b^	0.12	<0.01

a,b means within a row with different subscripts differ when P-value < 0.05.

### Correlation analyses

#### Correlation between muscle metabolomics and meat quality

**Figure 6:**
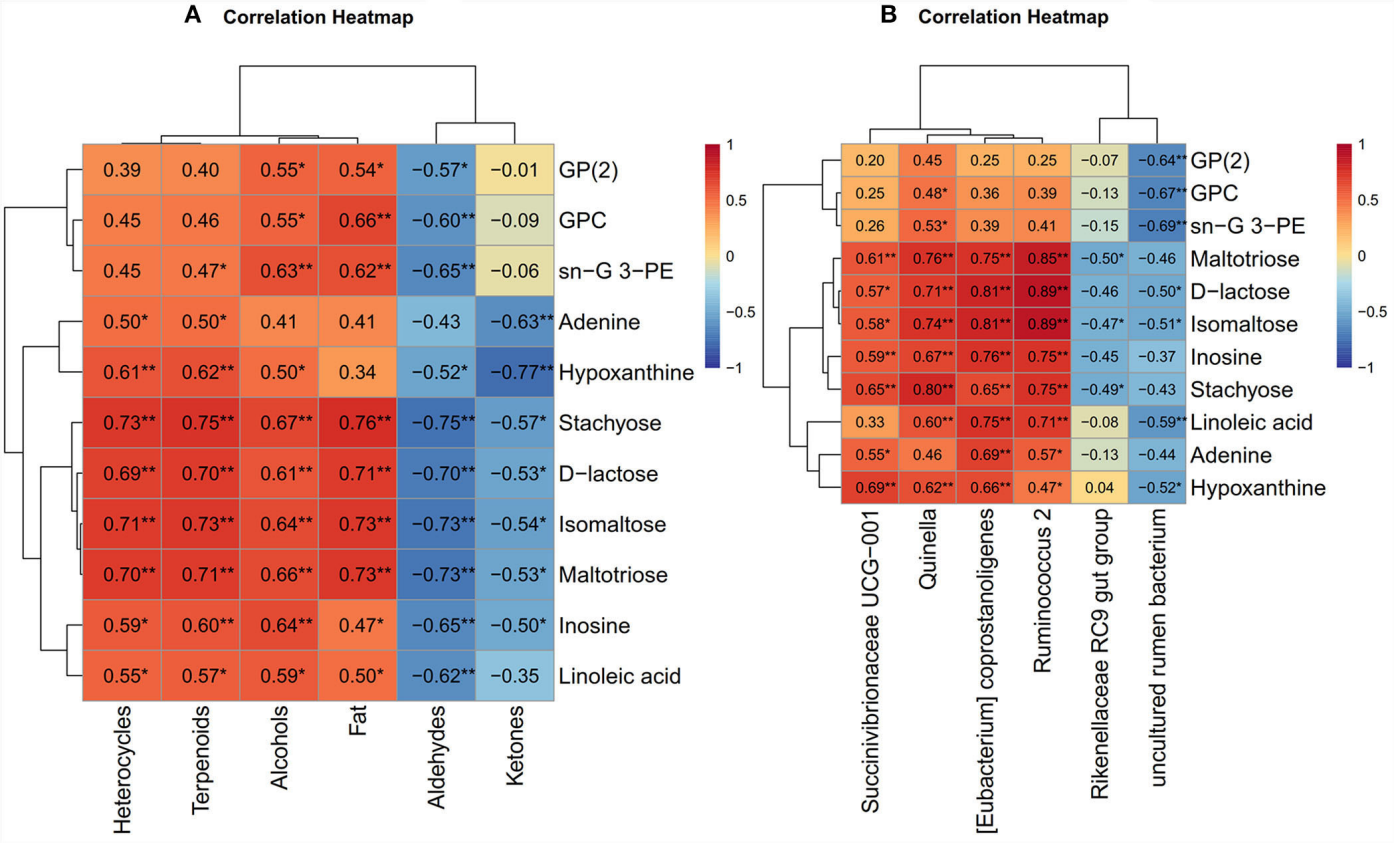
**(A**) The correlation heat map between meat quality parameters and muscle metabolomics analysis. **(B)** The correlation heat map between muscle metabolomics analysis and rumen bacteria. The color red and blue represent positive and negative correlations, respectively. *P < 0.05 and **P < 0.01. G: glycerol; PE: phosphoethanolamine; GP(2): glycerophosphate(2); GPC: glycerophosphocholine.

The correlation between the metabolism and quality of Black Tibetan sheep mutton fed on a diet with varying energy levels was evaluated using metabolomics and phenotypic data from the LL. We observed a significant association between muscle metabolism and meat quality (Figure 6A). In particular, the amount of fats in the LL positively correlated with the sn–glycerol 3-phosphoethanolamine, glycerophosphate (2), stachyose, isomaltose, D–lactose, maltotriose, and glycerophosphocholine levels. The levels of heterocycles, alcohols, and terpenoids were positively correlated with the adenine, hypoxanthine, inosine, stachyose, isomaltose, D–lactose, maltotriose, and linoleic acid levels, whereas the levels of aldehydes and ketones were negatively correlated with these metabolites. Moreover, aldehydes displayed a negative correlation with glycerophosphate (2), sn–glycerol 3-phosphoethanolamine, and glycerophosphocholin. However, the level of alcohols positively correlated with those of glycerophosphate (2), sn–glycerol 3-phosphoethanolamine, and glycerophosphocholin. Overall, the regulation of purine and carbohydrate metabolism (carbohydrate digestion and absorption, sucrose and starch metabolism, and galactose metabolism) significantly affects the levels of aldehydes, ketones, alcohols, heterocycles, and terpenoids in the LL. Regulation of the lipid metabolic pathways (ether lipid, glycerolipid, and glycerophospholipid metabolic pathways) significantly affects the levels of fat, alcohols, and aldehydes in the LL. In addition, carbohydrate metabolism affects fat content.

#### Correlation between muscle metabolomics and rumen bacteria

The correlation between muscle metabolomics and rumen bacteria was performed to explore the relationship between the level of metabolites in the LL and the composition of bacteria in the rumen of Black Tibetan sheep fed on diets with different energy levels. A similar correlation was observed between the level of muscle metabolites and the abundance of rumen bacteria (Figure 6B). Particularly, there was a positive correlation between the stachyose, isomaltose, D-lactose, maltotriose, linoleic acid, and inosine levels and the abundance of *Quinella, Ruminococcus 2*, and *coprostanoligenes (Eubacterium)*. There was also a positive correlation between hypoxanthine levels and the abundance of *Quinella* and *coprostanoligenes (Eubacterium)*, and between *coprostanoligenes (Eubacterium)* and adenine levels. Moreover, the levels of glycerophosphate (2), linoleic acid, glycerophosphocholine, and sn-glycerol 3-phosphoethanolamine negatively correlated with the abundance of uncultured rumen bacterium. The abundance of *Succinivibrionaceae UCG-001* positively correlated with the adenine, hypoxanthine, inosine, and stachyose levels.

## Discussion

The energy and nutrient levels of feeds significantly affect the carcass quality of sheep (18). Previous reports have shown that high concentrate diets increase the rumen microbes, enhance digestion, improve the feed conversion rate, and enhance the absorption of nutrients (19), attributed to the high energy and nutrient levels in feeds (2). We found that high-energy diets give the best carcass quality of Black Tibetan sheep, consistent with previous reports (20, 21).

The pH change affects the quality traits (e.g., color and WHC) of meat, and thus is a key indicator of meat quality (6). Hammelman et al. found that the WHC and texture of meat were connected with the rate of post-slaughter pH decline (22). A rapid decrease in pH causes muscle contraction and denaturation of proteins, deteriorating the WHC and texture. In the current study, the initial pH of meat in MS and LS was substantially higher than that in HS. However, the final pH, on the other hand, did not differ throughout the three groups. This showed that high dietary energy diets slowed down the rate of pH decline in the LL (within 24 h after slaughtering), which may explain better the WHC and texture of meat for sheep in the HS group relative to those from MS and LS. Wang et al. reported that increasing the dietary energy decreased the drip loss and pressing loss of mutton (23). Similarly, Jin et al. found that the cooking loss was lower for mutton of lambs fed high-energy feeds (24). These findings show that high energy level diets can improve the WHC of muscles. Nevertheless, before this study, the effect of high energy feeds on muscle texture, including hardness, adhesion, and chewiness, had not been reported.

The pH decline in muscles is caused by the accumulation of lactic acid produced by glycogen degradation. Meanwhile, the rate and extent of this process are jointly determined by the substrate content and the activities of key glycolytic enzymes in muscles (25). Rosenvold et al. discovered that the higher the glycogen content in muscles, the greater the post-slaughter pH decline in muscles (26). Interestingly, high-energy level diets increase muscle glycogen deposition (27). Furthermore, AMPK (adenosine monophosphate-activated protein Kinas, HS vs. LS) and PTS (phosphotransferase system, HS vs. MS) signaling pathways, both metabolism-related pathways, were downregulated and upregulated, respectively, in the HS group. AMPK indirectly increases glycolysis (28). AMPK is activated in hypoxic skeletal muscle, further activating phosphorylase kinase, which promotes the activities of glycogen phosphorylase and, thereby, glycogen decomposition (29). AMPK promotes the formation of fructose 2,6-diphosphate, an allosteric activator of phosphofructokinase-1 (the key rate-limiting enzyme of glycolysis), by promoting the phosphorylation of phosphofructokinase-2 (30). Inhibiting AMPK activation suppresses the glycolysis in muscles and, thus, is a feasible strategy for improving meat quality (31). Notably, the transfer and phosphorylation of the carbon source in PTS reduce glucose phosphorylation, reducing the concentration of cAMP (cyclic adenosine monophosphate). Meanwhile, this inhibits the APK (cAMP-dependent protein kinase) signaling pathway and the phosphorylation of inactive glycogen phosphorylase, decreasing glycogen metabolism (32). Additionally, Chen et al. found that dietary magnesium supplementation reduces the formation of cAMP and, thereby, glycolysis (33). In this study, we speculated that downregulating the AMPK signaling pathway inhibits the activity of AMPK, whereas upregulating PTS reduces the concentration of cAMP and subsequently modulates the decline of pH in the LL. Overall, it is suggested that high-energy level diets regulate the rate of pH decline in the LL by inhibiting glycolysis and thus improving muscle WHC and texture of Black Tibetan sheep. In particular, these events are achieved by upregulating PTS (HS vs. MS) and downregulating AMPK (HS vs. LS) signaling pathways. However, more evidence is needed to support this theory.

Meat nutrient quality indicators mainly rely on parameters such as moisture content, the proportion of ash, protein, and fats, and the composition of FAs and AAs. In this study, we revealed that dietary energy levels impact the nutritional value of Black Tibetan sheep mutton based on fat content, which is consistent with previous research (34, 35). Mushi et al. found that meat's fat content positively correlates with dietary energy levels (34). Similarly, Majdoub-Mathlouthi et al. found that high dietary energy levels promote fat deposition in beef (35). Unexpectedly the higher fat deposition in HS, lower in MS, and medium in LS was detected in our findings. The discrepancy between our findings and previous studies may be caused by the different NDF and ADF ratios in the diets (36). Notably, an increase in fat deposition in muscles might be associated with increased metabolism in these tissues (37). In the previous research, we discovered that indoor feeding increased the content of fat in sheep's LL by regulating the lipolysis in the adipocyte pathways of muscles (10). Moreover, previous reports show that the increased carbohydrate metabolism could generate more energy and substrate for the host for the synthesis of fat in muscles (38, 39). In this study, we found a strong correlation between the fat content and the metabolism of lipids (ether lipid, glycerolipid, and glycerophospholipid) and carbohydrates (carbohydrate digestion and absorption, sucrose and starch, and galactose). As such, these processes are potential indicators of fat deposition. Furthermore, optimal fat deposition can improve meat's juiciness, palatability, and flavor. The breakdown of myofibrillar proteins and connective tissue may be responsible for the reduction in cooking and drip loss of muscles (40). Interestingly, myofibrils and connective tissues have a strong relationship with the distribution and composition of proteins and lipids in muscles (40). The optimal fat deposition can loosen muscle tissue, which improves water retention in meat. Therefore, the better WHC and texture of meat for sheep in the HS group might be attributed to the fat deposition. However, the variation of a muscle phenotypic trait might be affected by multiple factors and their interaction (Starkey et al. 2015, Starkey et al. 2017). As such, more studies on the rate of post-slaughter pH decline, fat content, protein degradation, and myofibril structure are needed to predict the factors affecting muscle WHC and texture.

The volatile flavor is an important parameter for assessing meat quality. Most of the VOCs detected in sheep mutton included aldehydes, alcohols, and ketones, consistent with the previous studies (41, 42). Aldehydes are an important group of fragrance and flavor compounds. Most aldehydes impart a good aroma at low concentrations. It is reported that the increased concentrations of certain aldehydes above a certain level can cause rancidity or unpleasant odors in meat (43). For example, hexanal gives meat the apple, leaf, and delicate aromas; heptanal imparts the nutty flavor and a fruity aroma. n-Nonanal has a distinct fatty and rancid flavor, whilst pentanal has an unpleasant spicy smell and the characteristic oil and wax scent (44). Benzaldehyde is associated with an unpleasant taste (11). In addition, compared with aldehydes, alcohols exert a lesser effect on the mutton flavor. Nevertheless, at high concentrations, alcohols impart vanilla, woody, and high-fat flavors to meat products (45). Notably, ketones, a class of VOCs with floral, fruity, and creamy aromas, influence the overall flavor of meat products (46). Ethyl acetate is another compound low in concentration in meat that improves the mutton flavor by imparting fruity and winey aromas (47). Pyrazines are heterocyclic substances with a strong aroma (48). Terpenoids are mainly found in plants but can be transferred to animal meat through direct digestion (49). Accumulation of limonene imparts a fresh fruity flavor to meat products (50). We found that feeding sheep with high-energy diets increases the VOC content (alcohols, ethyl acetate, limonene, and 2,3-dimethyl-5-ethylpyrazine) in the LL, which imparts good flavor to mutton. The low content of certain VOCs (hexanal and heptanal) improved the flavor of mutton in the HS group, whereas high concentrations of valeraldehyde, nonanal, and benzaldehyde had a negative effect on the mutton flavor in MS and LS.

Lipids and FAs are the major sources of VOCs in meat (51). Meanwhile, aldehydes, alcohols, and ketones are the main oxidation and degradation products of lipids and FAs (52). Accordingly, there was a strong correlation between the content of VOCs and the oxidation of lipids and FAs in muscles. In addition, we found that the levels of dietary energy had no influence on the amount of FA in meat, which is consistent with previous research (5, 35). High energy diets increased the amount of linoleic acid but medium energy diets reduced that of the lipid-derived metabolites, including glycerophosphocholine, sn-glycerol 3-phosphoethanolamine, and glycerophosphate (2), based on muscle metabolomes. Interestingly, linoleic acid and sn-glycerol 3-phosphoethanolamine are closely related to terpenoids. Terpenoids are antioxidants in meat (53), whereas high-level aldehydes in meat are caused by low content of naturally occurring antioxidants (54). Therefore, terpenoids only detected in HS could explain the lower aldehyde content and higher alcohol content in mutton of Black Tibetan sheep fed on high-energy feeds because they protect against oxidation and degradation of lipids.

Amino acids also greatly affect the taste and flavor of mutton. Certain AAs strongly participate in Maillard reaction and Strecker degradation to produce desired VOCs (55). Some AAs are the main determinants of the taste of mutton. Glycine, alanine, serine, and threonine give mutton a sweet taste, whereas aspartate and glutamate give the meat an umami taste, all likable by consumers (56). Several studies show that except for some flavor AAs, dietary energy levels do not affect the content of most AAs (1, 5), consistent with our findings. High levels of glycine and serine in the LL of the MS group relative to the LS group possibly enhanced the sweetness of the meat. While no effect of dietary energy was observed on the aspartate and glutamate levels, medium energy diets increased the asparagine and glutamine content in mutton relative to sheep fed on low energy diets. More importantly, medium energy diets increased the metabolic rate of D-glutamate and D-glutamine and the production of several metabolites (L-glutamine, D-glutamine, D-pyroglutamic acid, and glutamine). This might promote the synthesis of glutamate, which gives mutton the umami taste. Thus, we hypothesized that the MS group's mutton was sweeter than the LS group's sheep, whereas that of sheep in the HS group was in between. However, further analyses are needed to validate this hypothesis.

Increasing and reducing sugar levels is another key way of enhancing the flavor of the meat. They give the meat a distinctive flavor through the Maillard reaction (57). Similarly, the degradation of nucleotides, such as the release of ribose phosphate, adenosine and inosine, and free ribose, gives the meat an additional flavor, analyzed through the Maillard reaction (58). Also, some VOCs are produced through the interaction of the Maillard reaction and lipid oxidation (59). Perhaps, reducing sugars from carbohydrate metabolism and nucleotide degradation creates an environment that promotes the Maillard reaction. The reducing sugars, in particular, interact with lipid degradation products, resulting in the production of more VOCs after cooking, which could explain the high number of VOCs in the HS group compared to others. A recent study revealed that the levels of most VOCs in yak meat are influenced by different metabolic components, such as 6-phosphate-glucose, dehydroshikimate, stearidonic acid, etc., and different metabolic pathways, including amino acid metabolism (enrichment) pathways, and starch and sucrose (prediction) among others (11). In the present study, we found that nucleotide-derived and carbohydrate-derived metabolites regulate the production of aldehydes, alcohols, ketones, terpenoids, and heterocycles in mutton. Besides, aldehydes and alcohols are also regulated by lipid-derived metabolites. Overall, the meat flavor of Black Tibetan sheep can be significantly enhanced by regulating the metabolism of lipids (glycerophospholipid, ether lipid, and glycerolipid metabolism), carbohydrates (sucrose and starch, and galactose metabolism digestion and absorption), and purines in the LL.

**Figure 7:**
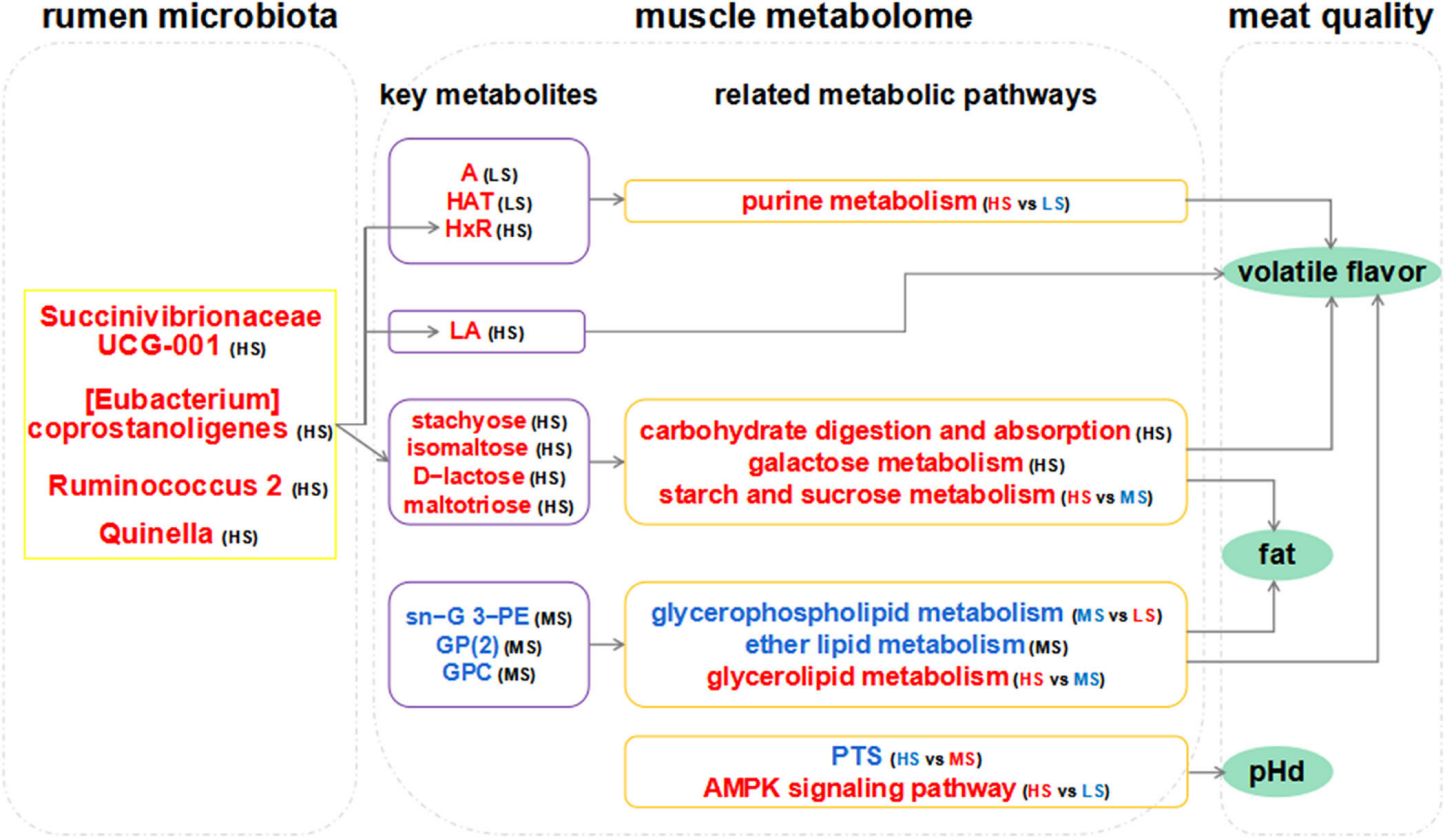
Hypothesized scheme pathways and potential mechanisms related to the changes in rumen microbiota, muscle metabolome, and meat quality. Metabolites and bacteria in blue and red indicate them downregulated and upregulated significantly in each comparison, respectively. The gray line denotes the regulatory pathways of meat quality. A: adenine; HAT: hypoxanthine; HxR: inosine; PE: phosphoethanolamine; GP(2): glycerophosphate(2); GPC: glycerophosphocholine; LA: linoleic acid; G: glycerol. pHd: the extent of pH decline (within 24 h after slaughter).

The pH value, ammonia-N level, and VFA concentration are the main parameters of rumen fermentation. A high-energy diet can increase the concentration of VFAs and, subsequently, pH reduction in the rumen (23), consistent with our findings. This maybe due to a high-energy diet containing a large amount of non-fibrous carbohydrates, which can be rapidly fermented by rumen microorganisms. Nonetheless, the composition of rumen microbiota determines rumen fermentation types (e.g., amylolytic, proteolytic, and cellulolytic), and the products of fermentation impact the traits of the host meat (60). *Firmicutes* and *Bacteroidetes* were the primary phyla altered by dietary energy levels in this study, and they were highly correlated with carbohydrate metabolism, which is consistent with previous findings (61, 62). As such, the high-energy level diets influenced the composition of rumen microbiota and thus, the metabolism in the rumen. This result eventually produced the difference in metabolic levels in the muscle due to a direct correlation amid the abundance of four species at the genus level (*Quinella, Ruminococcus 2, Succinivibrionaceae UCG-001, and (Eubacterium) coprostanoligenes*) and the content of the products of carbohydrate metabolism (stachyose, isomaltose, D-lactose, and maltotrios) in the LL. The high energy supply promoted the rapid proliferation of these bacteria, which in turn degrade carbohydrates (38). This process provides substrates and energy to fat synthesis and VOCs formation in the LL and thus, generates better mouthfeel, nutrition, and flavor of Black Tibetan sheep mutton. Furthermore, we speculated on the potential mechanisms by which rumen microbiota composition affects muscle metabolism and meat quality in this study (Figure 7). However, further studies are needed to verify the mechanism of the effect of the composition of rumen microbiota on the quality and flavor of mutton.

## Conclusion

A high-energy diet improves the carcass and meat quality of Black Tibetan sheep. Also, a high energy supply alters the production of VOCs which gives meat better flavor. However, dietary energy levels, on the other hand, have essentially minimal influence on the AA and FA profiles in the LL. The quality and flavor of Black Tibetan sheep mutton are influenced by dietary energy levels, which regulate the metabolism in muscular tissues. Upregulation of PTS and downregulation of the AMPK signaling pathways suppress the rapid decline of pH. High lipid (glycerophospholipid, glycerolipid, and ether lipid) and carbohydrate (galactose, starch, and sucrose, and the digestion and absorption of carbohydrates) metabolisms are potential indicators of fat deposition. Together with purine metabolism, lipid and carbohydrate metabolisms affect the formation of VOCs. Correlation analyses show that high-energy feed influences the deposition of key metabolites (stachyose, isomaltose, D-lactose, maltotriose, linoleic acid, and inosine) and regulates related metabolic pathways by altering the composition of rumen microbiota (increasing the abundance of *Quinella, Ruminococcus 2, (Eubacterium) coprostanoligenes*, and *Succinivibrionaceae UCG-001*) in Balck Tibetan sheep's LL. In the end, these processes improve the quality and flavor of Black Tibetan sheep mutton.

## Data availability statement

The authors acknowledge that all relevant data presented in this study are contained within the article/Supplementary material, further inquiries can be directed to the corresponding author/s.

## Ethics statement

The animal study was reviewed and approved by the study was carried out at the Black Tibetan Sheep Breeding Center in Guinan County, Qinghai Province approved by the Animal Ethics Committee of Qinghai University (QUA-2020-0709). Written informed consent was obtained from the owners for the participation of their animals in this study.

## Author contributions

XZ and LH: conceptualization, data curation, formal analysis, investigation, methodology, software, validation, writing—original draft, and writing—review & editing. SHAR, SH, LG, SSu, and ZW: conceptualization, software, validation, data curation, formal analysis, investigation, methodology, software, validation, writing—original draft, and writing—review & editing. BY, ZY, JS-G, AE-S, AA, and MA: visualization, software, validation, data curation, formal analysis, writing—review & editing, methodology, and funding acquisition. MS, SSa, and BA: writing—review & editing and methodology. All authors have read and agreed to the published version of the manuscript.

## Funding

This research was funded by Evaluation and Analysis of Nutritional Value of Black Tibetan Sheep and Research on Development of Series Products Funds of Qinghai Province (2020-GN-119). The current work was funded by Taif University Researchers Supporting Project number (TURSP-2020/75), Taif University, Taif, Saudi Arabia.

## Conflict of interest

The authors declare that the research was conducted in the absence of any commercial or financial relationships that could be construed as a potential conflict of interest.

## Publisher's note

All claims expressed in this article are solely those of the authors and do not necessarily represent those of their affiliated organizations, or those of the publisher, the editors and the reviewers. Any product that may be evaluated in this article, or claim that may be made by its manufacturer, is not guaranteed or endorsed by the publisher.
