# Supplementary figures and images for: Metabolomics approach reveals high energy diet improves the quality and enhances the flavor of black Tibetan sheep meat by altering the composition of rumen microbiota
Source: Front Nutr. 2022 Aug 10;9:915558. doi: 10.3389/fnut.2022.915558 (PMC9405419; doi:10.3389/fnut.2022.915558)

Scores (PLS-DA)

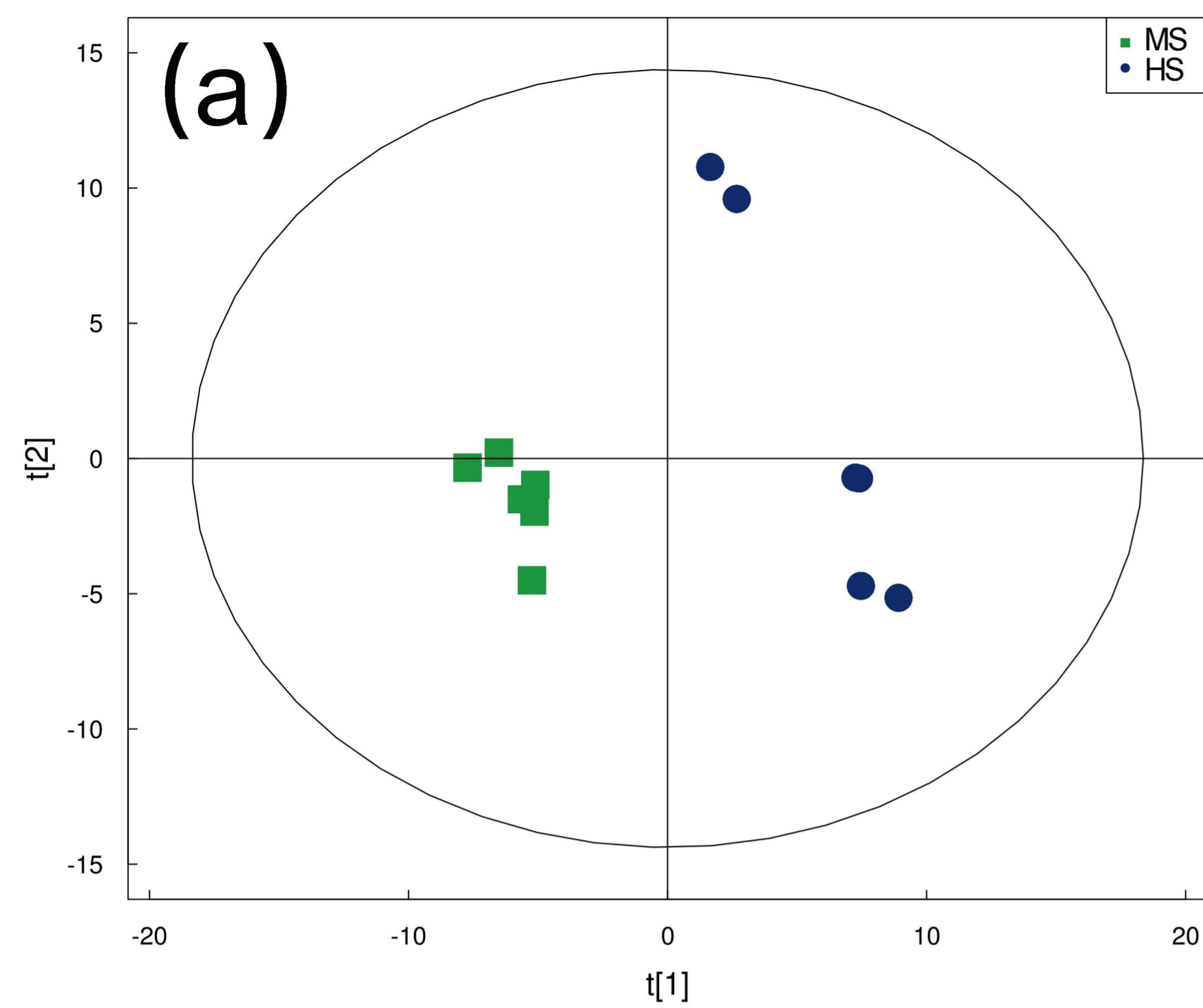

Scores (PLS-DA)

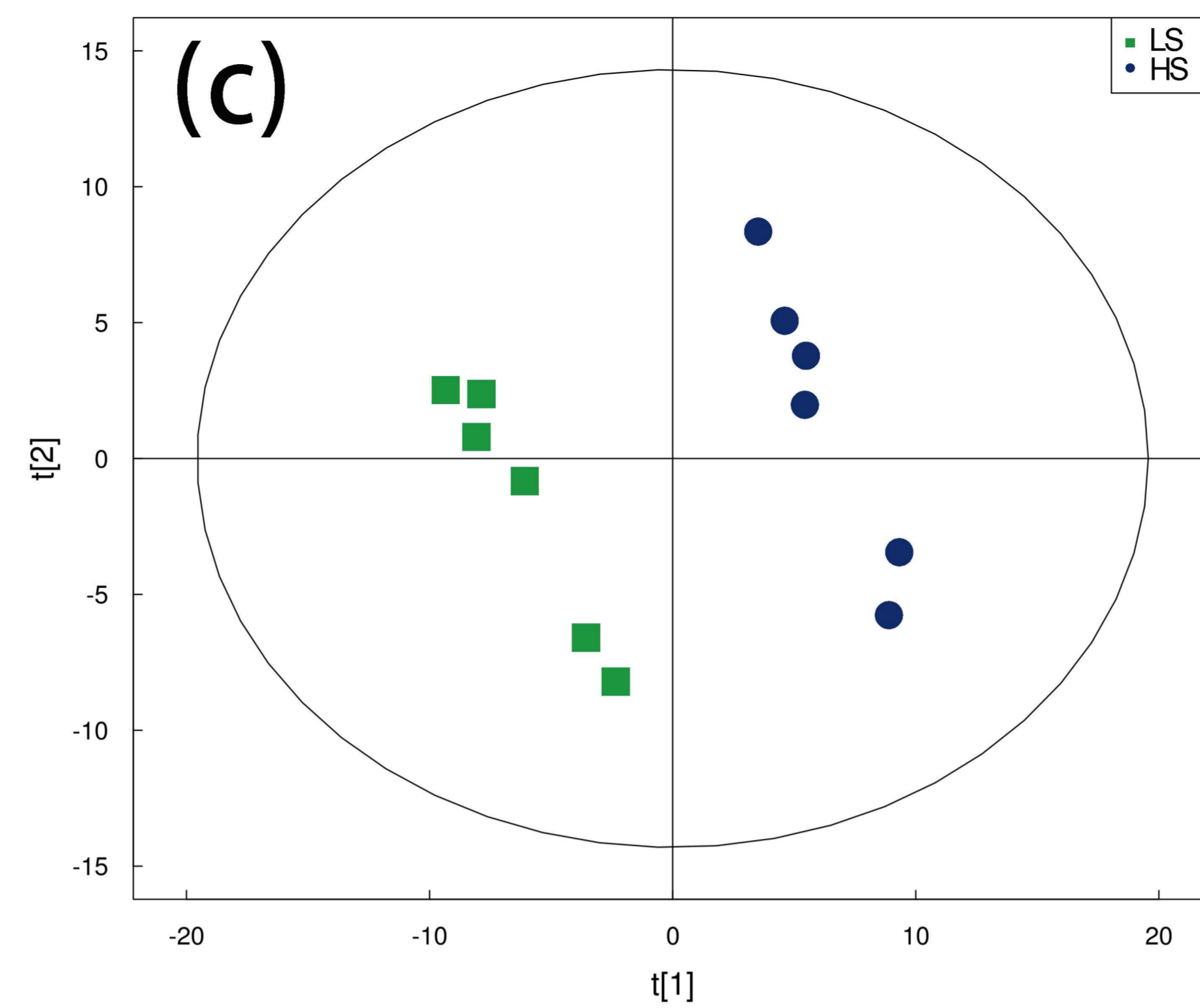

Scores (PLS-DA)

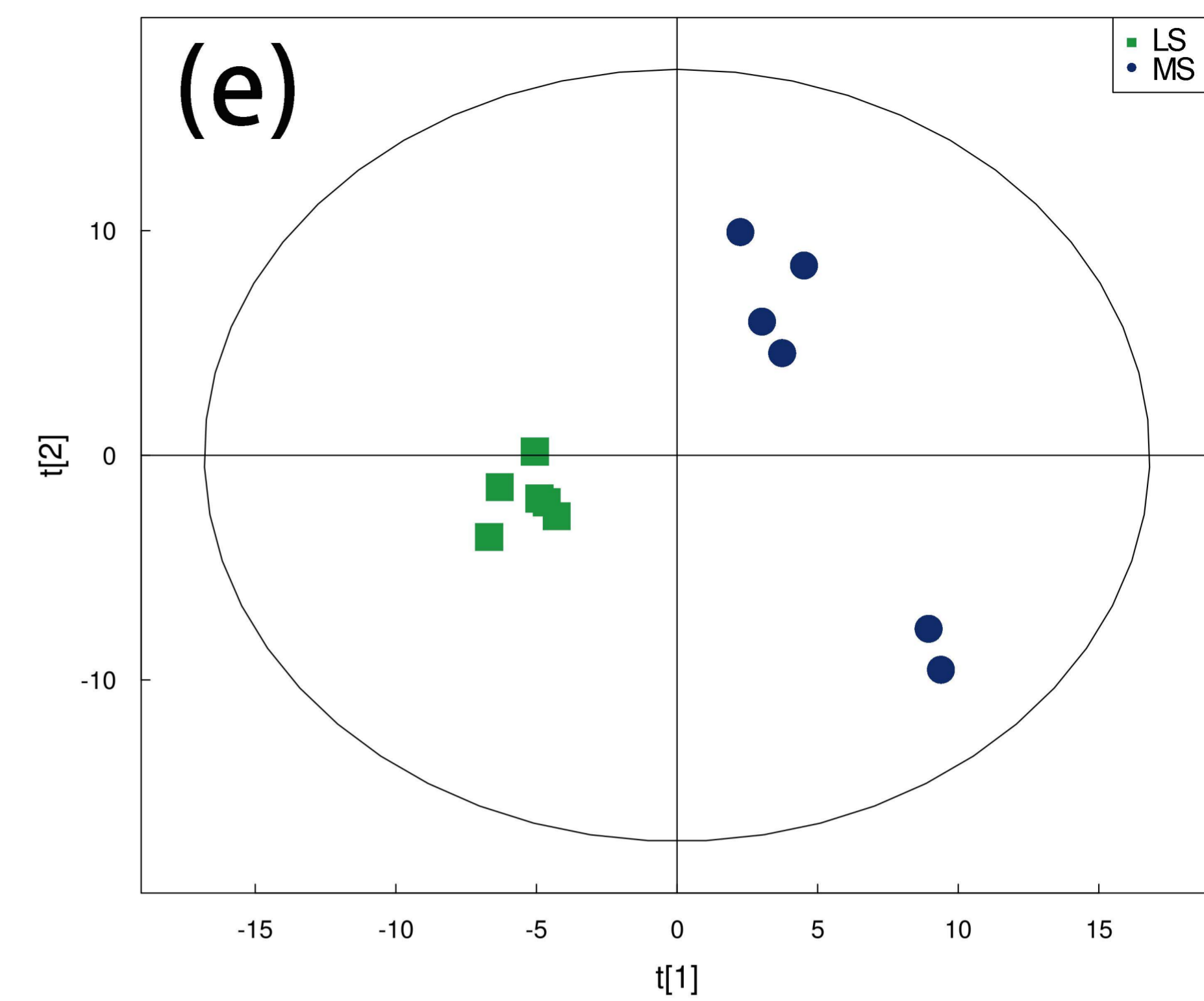

Scores (PLS-DA)

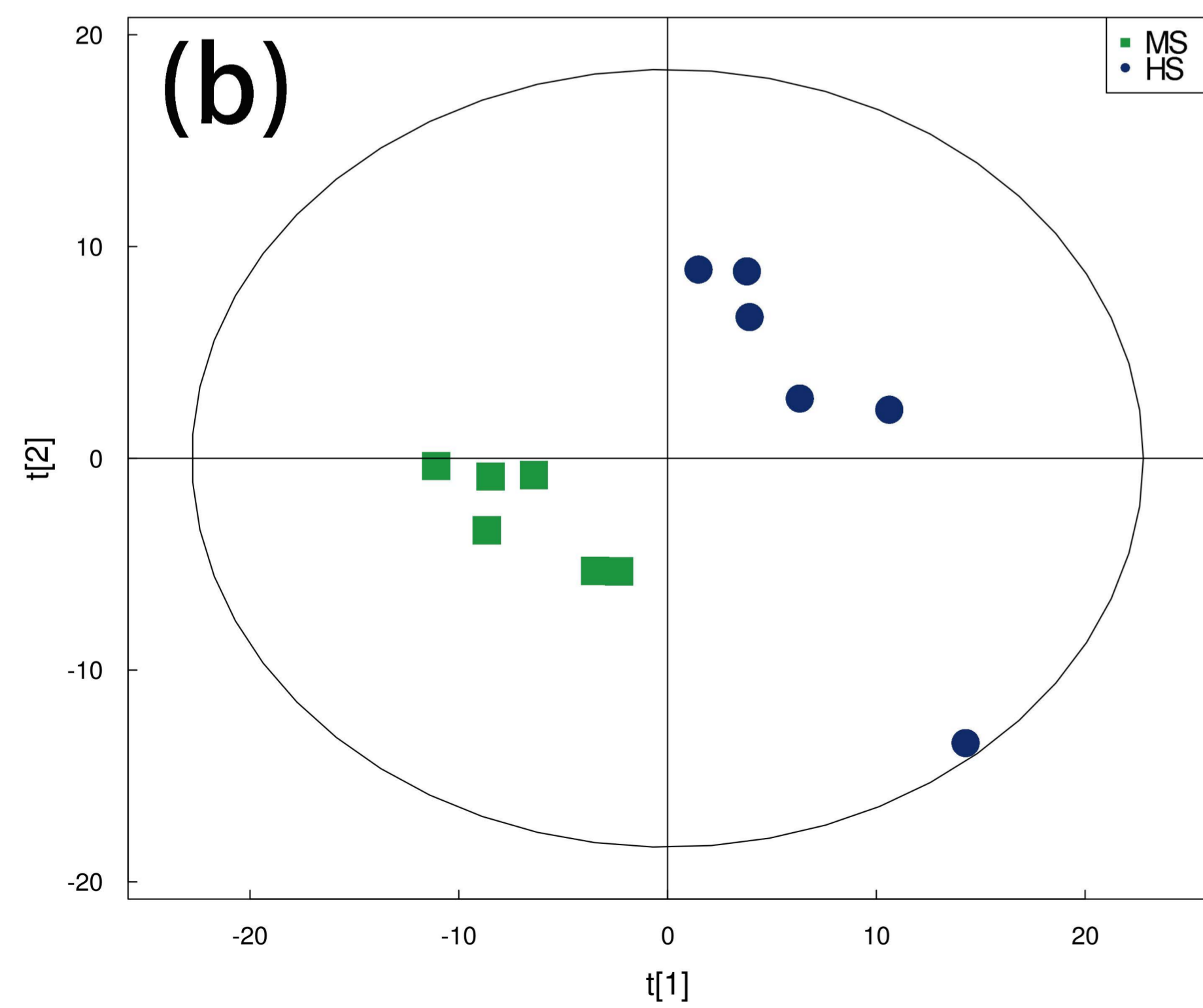

Scores (PLS-DA)

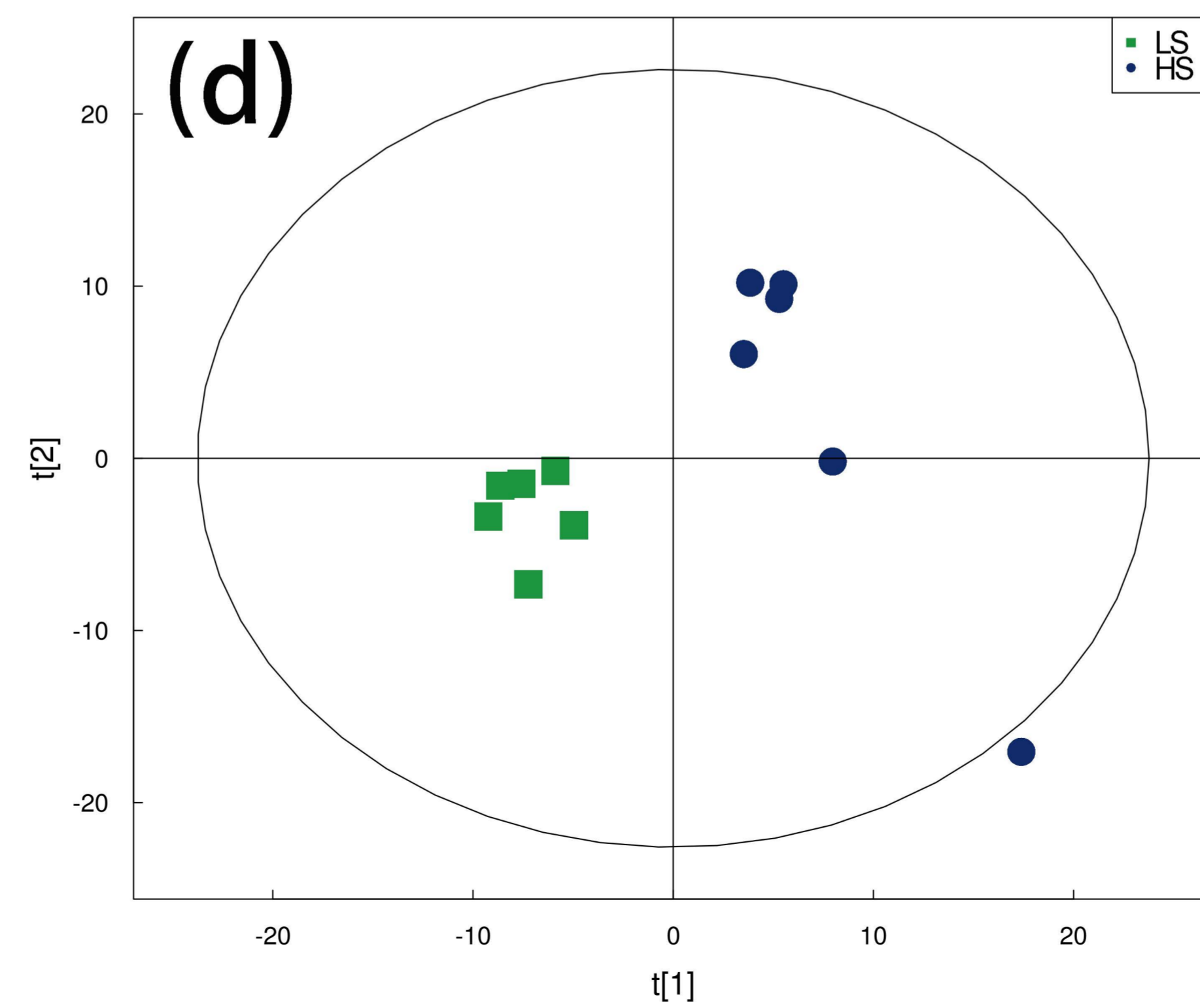

Scores (PLS-DA)

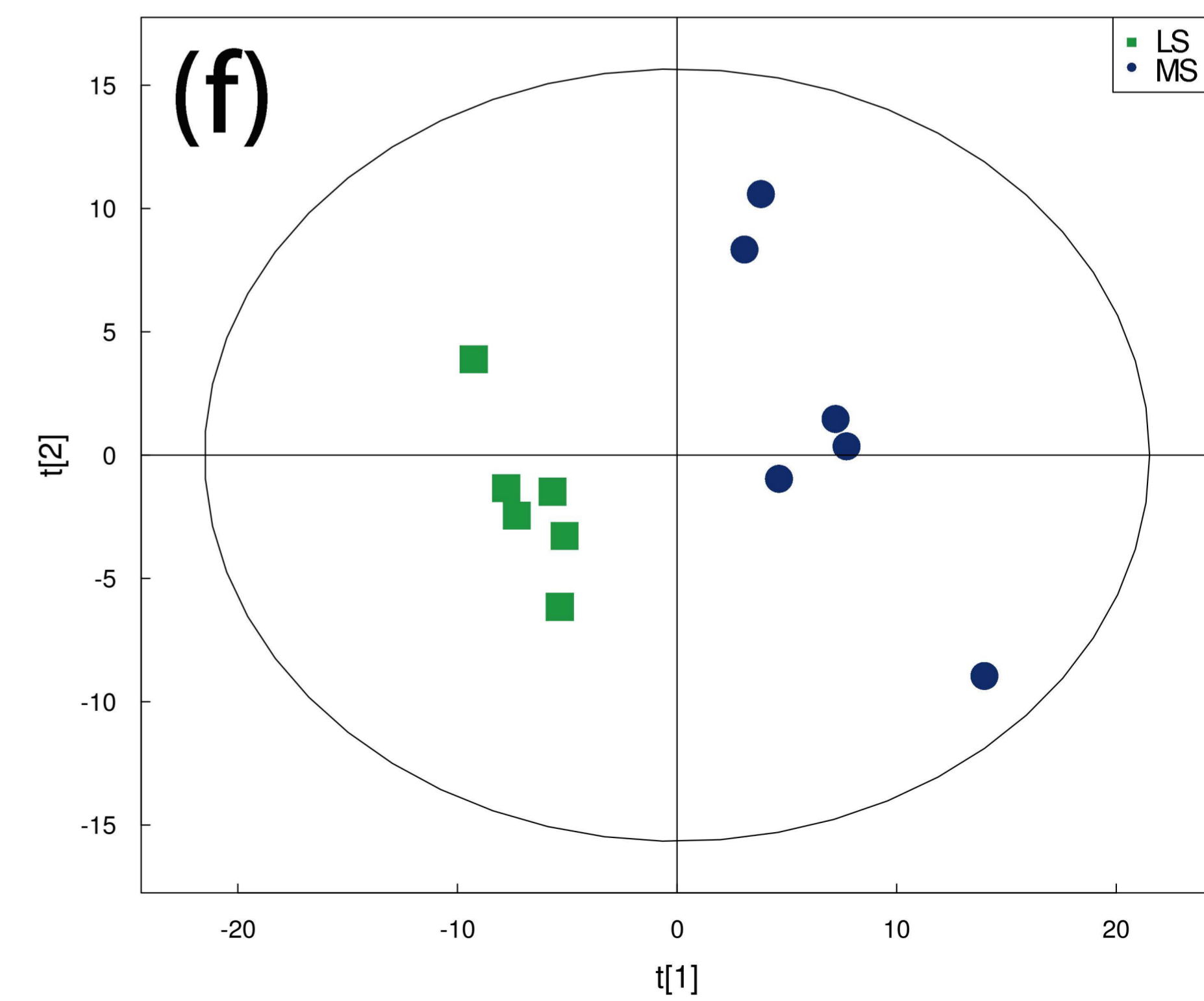

Supplement: Supplementary file 1 [file Image_1.pdf]

Scores (OPLS-DA)

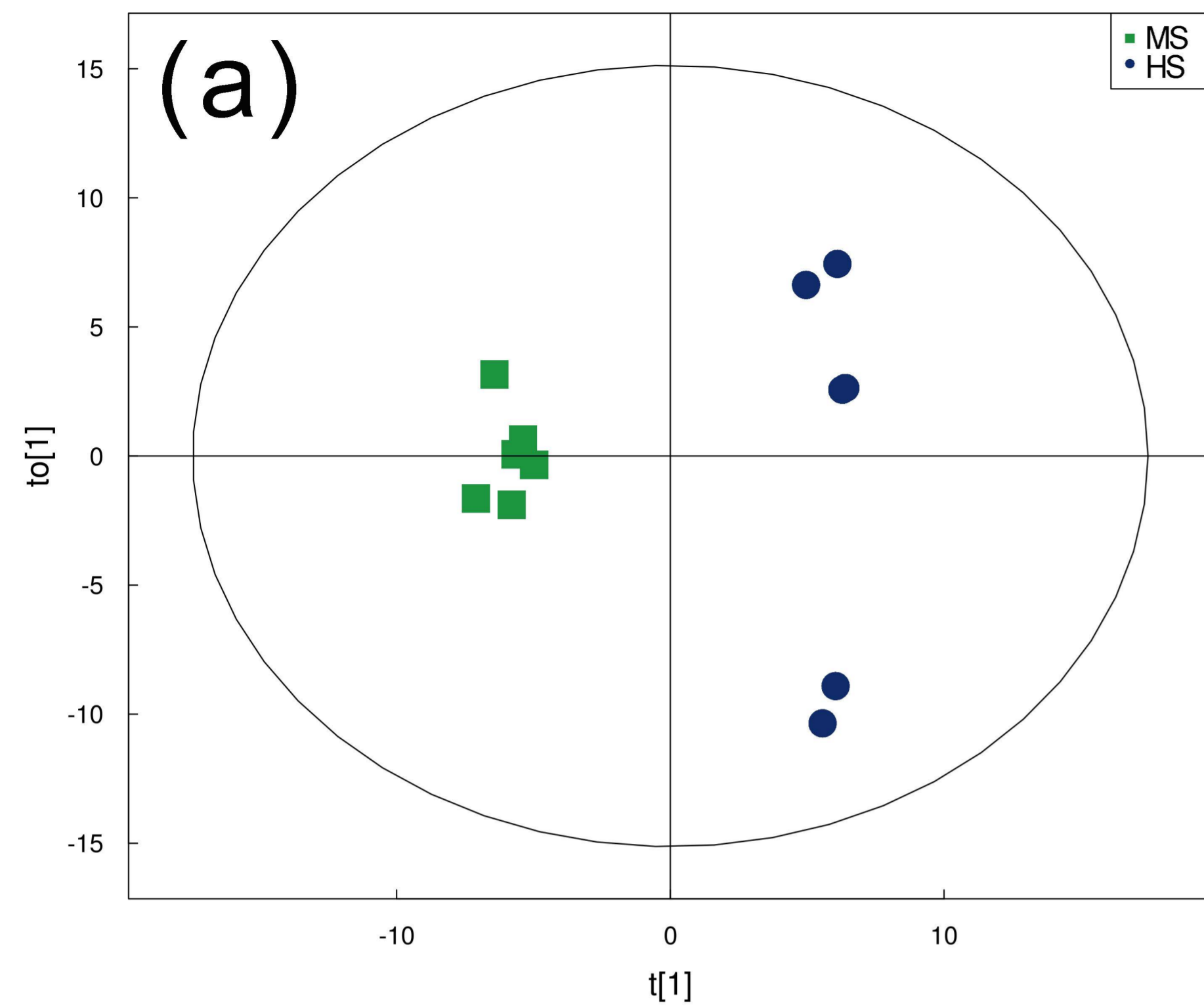

Scores (OPLS-DA)

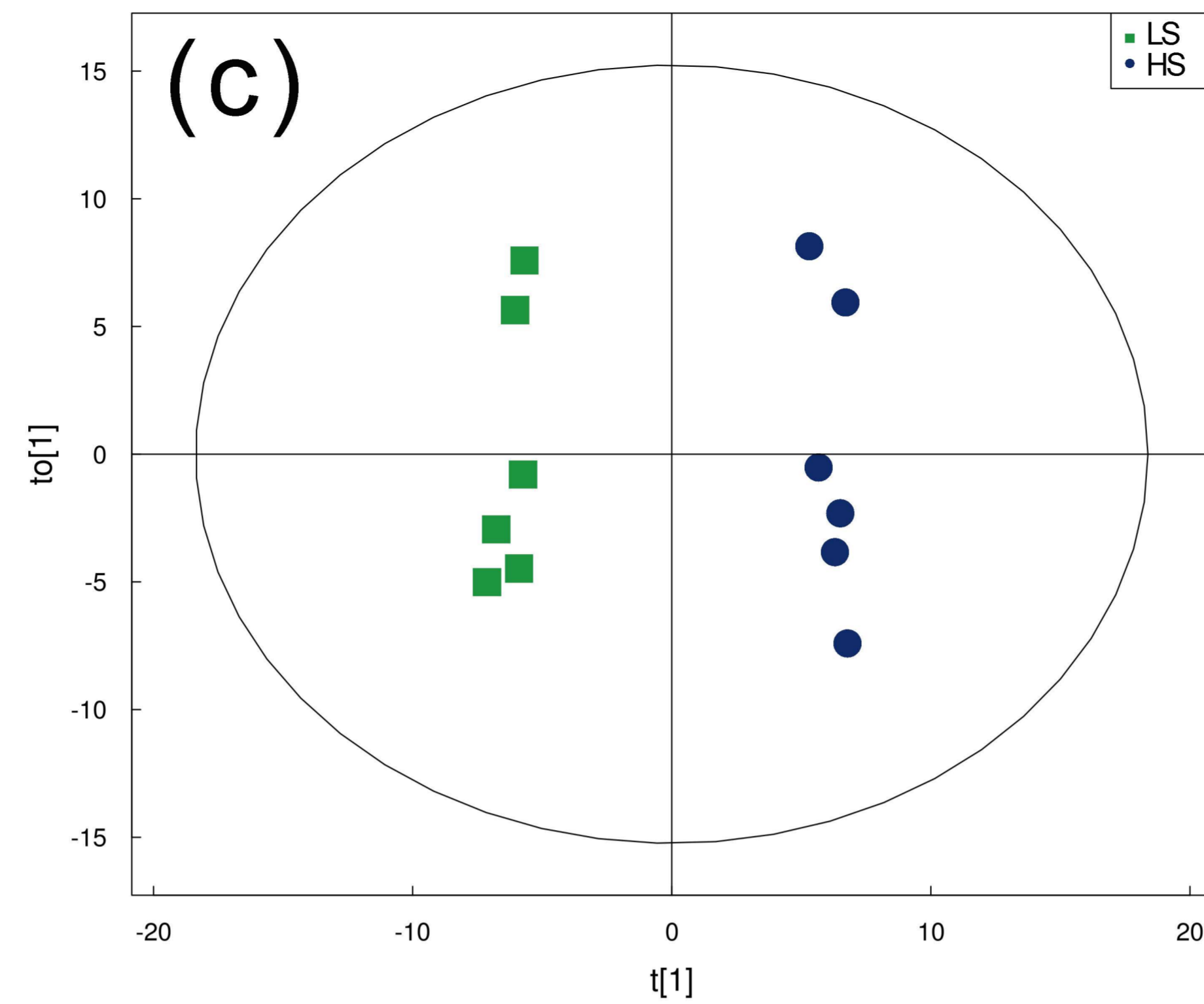

Scores (OPLS-DA)

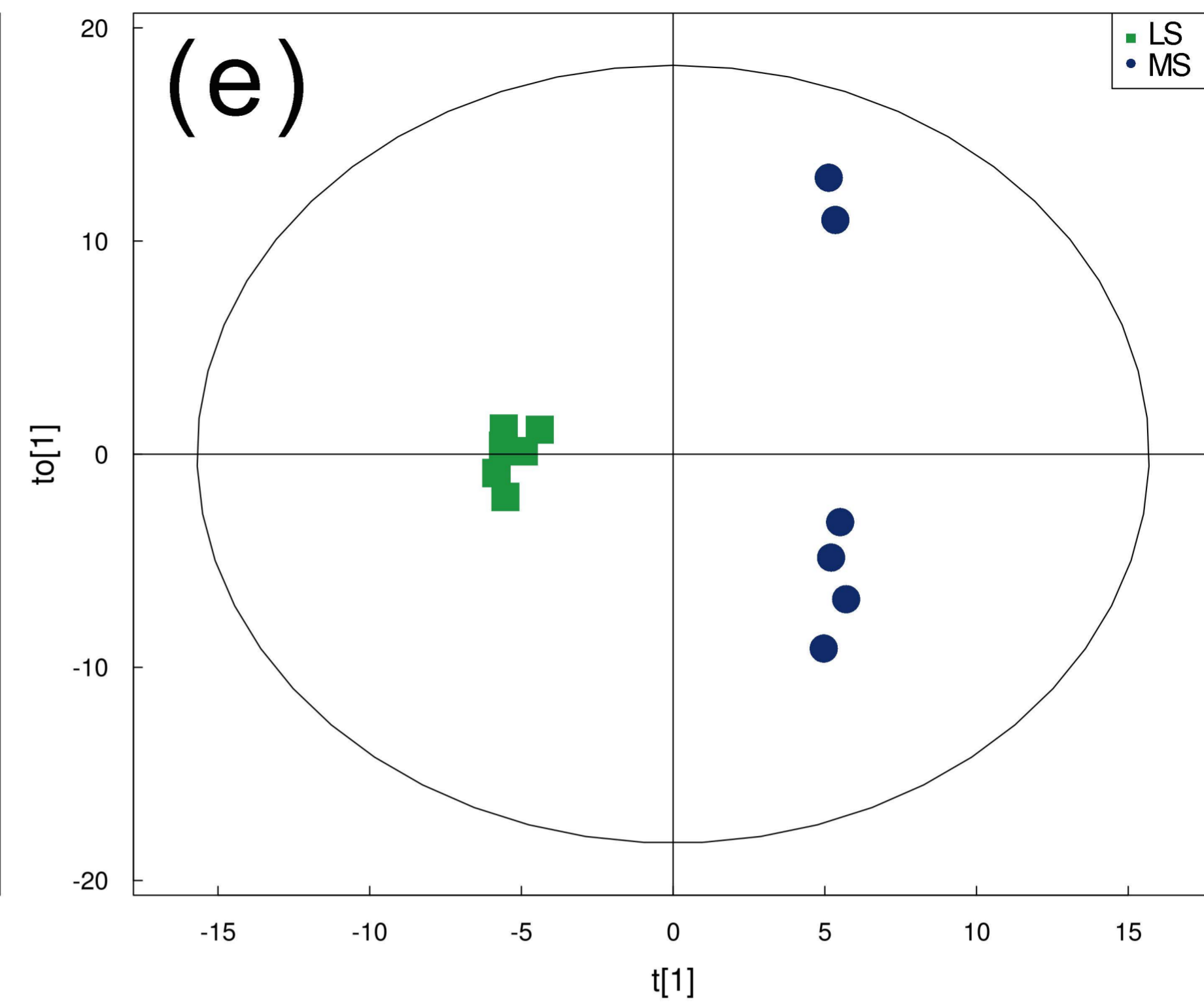

Scores (OPLS-DA)

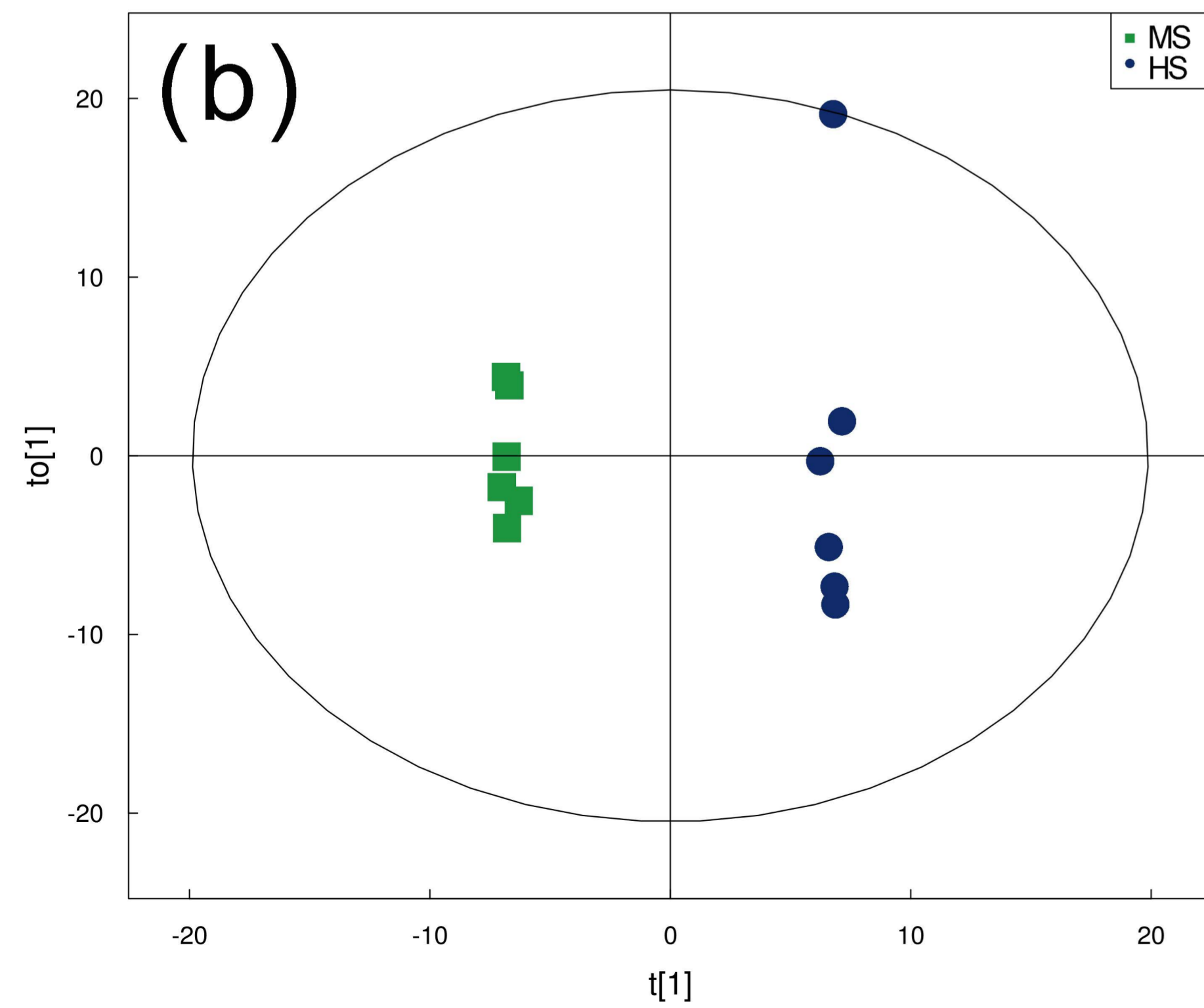

Scores (OPLS-DA)

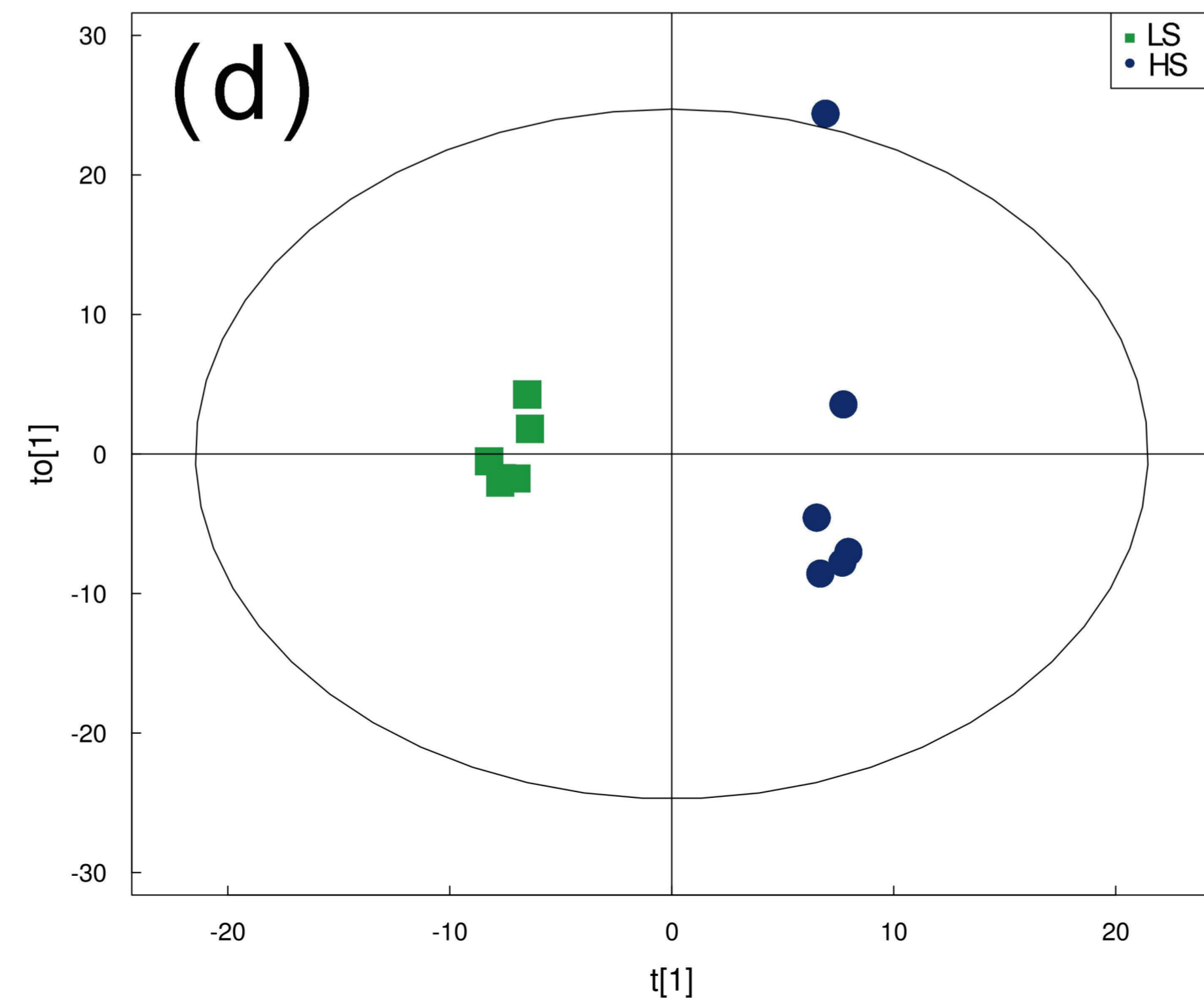

Scores (OPLS-DA)

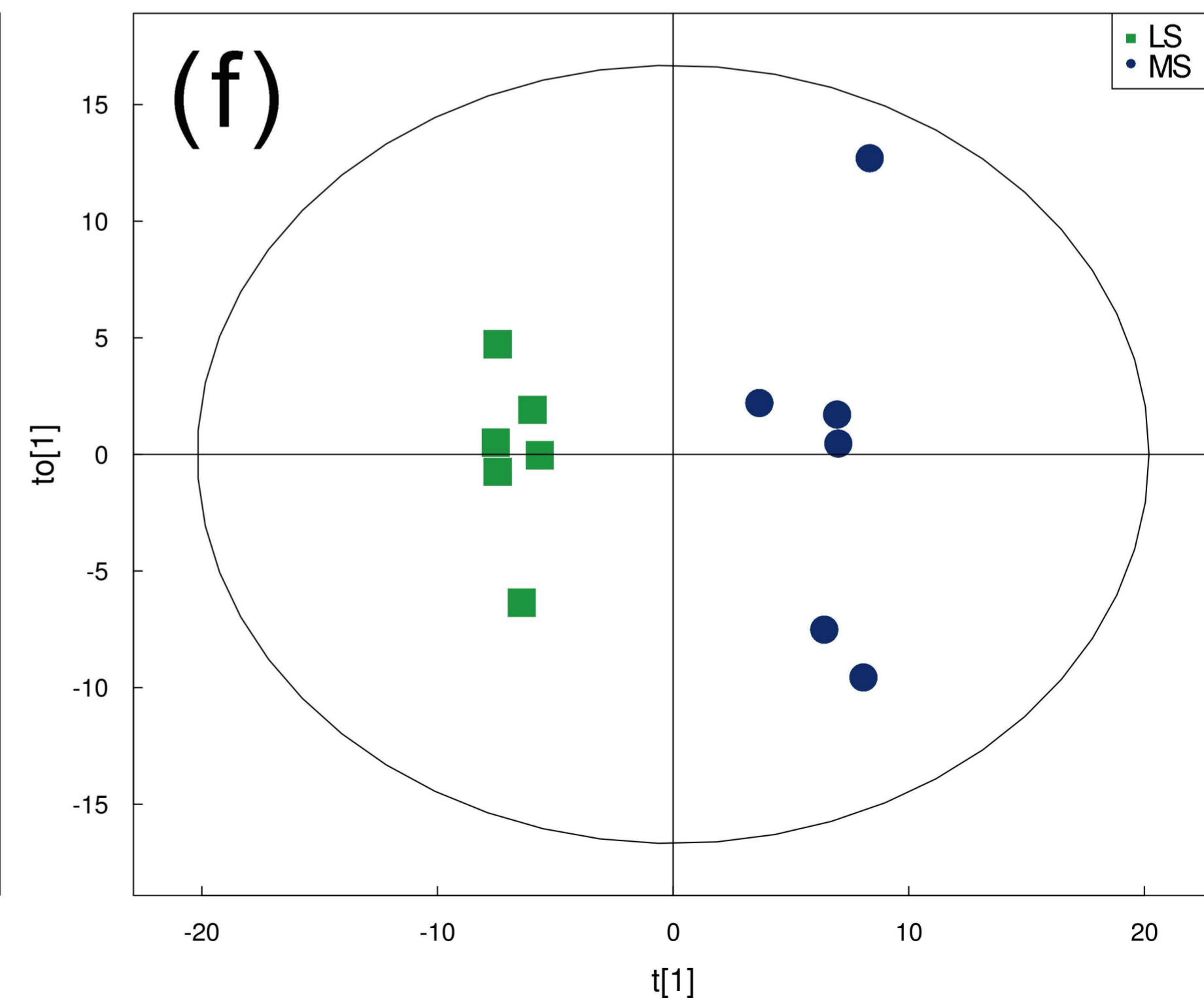

Supplement: Supplementary file 2 [file Image_2.pdf]
